# SARS-CoV-2 Coronavirus Spike Protein-Induced Apoptosis, Inflammatory, and Oxidative Stress Responses in THP-1-Like-Macrophages: Potential Role of Angiotensin-Converting Enzyme Inhibitor (Perindopril)

**DOI:** 10.3389/fimmu.2021.728896

**Published:** 2021-09-20

**Authors:** Tlili Barhoumi, Bandar Alghanem, Hayat Shaibah, Fatmah A. Mansour, Hassan S. Alamri, Maaged A. Akiel, Fayhan Alroqi, Mohammad Boudjelal

**Affiliations:** ^1^Medical Research Core Facility and Platforms (MRCFP), King Abdullah International Medical Research Center/King Saud bin Abdulaziz University for Health Sciences (KSAU-HS), King Abdulaziz Medical City (KAMC), National Guard Health Affairs (NGHA), Riyadh, Saudi Arabia; ^2^Department of Clinical Laboratory Sciences, King Saud bin Abdulaziz University for Health Sciences, Riyadh, Saudi Arabia; ^3^Department of Human and Molecular Genetics, School of Medicine, Virginia Commonwealth University, Richmond, VA, United States; ^4^Department of Pediatrics, King Abdulaziz Medical City, King Abdullah Specialized Children’s Hospital, Riyadh, Saudi Arabia

**Keywords:** SARS-CoV-2, spike protein, monocyte/macrophages, inflammation, angiotensin-converting enzyme inhibitor, ROS, HUVEC cells

## Abstract

A purified spike (S) glycoprotein of severe acute respiratory syndrome-related coronavirus 2 (SARS*-*CoV*-*2) coronavirus was used to study its effects on THP-1 macrophages, peripheral blood mononuclear cells (PBMCs), and HUVEC cells. The S protein mediates the entry of SARS-CoV-2 into cells through binding to the angiotensin-converting enzyme 2 (ACE2) receptors. We measured the viability, intracellular cytokine release, oxidative stress, proinflammatory markers, and THP-1-like macrophage polarization. We observed an increase in apoptosis, ROS generation, MCP-1, and intracellular calcium expression in the THP-1 macrophages. Stimulation with the S protein polarizes the THP-1 macrophages towards proinflammatory futures with an increase in the TNFα and MHC-II M1-like phenotype markers. Treating the cells with an ACE inhibitor, perindopril, at 100 µM reduced apoptosis, ROS, and MHC-II expression induced by S protein. We analyzed the sensitivity of the HUVEC cells after the exposure to a conditioned media (CM) of THP-1 macrophages stimulated with the S protein. The CM induced endothelial cell apoptosis and MCP-1 expression. Treatment with perindopril reduced these effects. However, the direct stimulation of the HUVEC cells with the S protein, slightly increased HIF1α and MCP-1 expression, which was significantly increased by the ACE inhibitor treatment. The S protein stimulation induced ROS generation and changed the mitogenic responses of the PBMCs through the upregulation of TNFα and interleukin (IL)-17 cytokine expression. These effects were reduced by the perindopril (100 µM) treatment. Proteomic analysis of the S protein stimulated THP-1 macrophages with or without perindopril (100 µM) exposed more than 400 differentially regulated proteins. Our results provide a mechanistic analysis suggesting that the blood and vascular components could be activated directly through S protein systemically present in the circulation and that the activation of the local renin angiotensin system may be partially involved in this process.

**Graphical:**

Suggested pathways that might be involved at least in part in S protein inducing activation of inflammatory markers (red narrow) and angiotensin-converting enzyme inhibitor (ACEi) modulation of this process (green narrow).

**Graphical Abstract:**
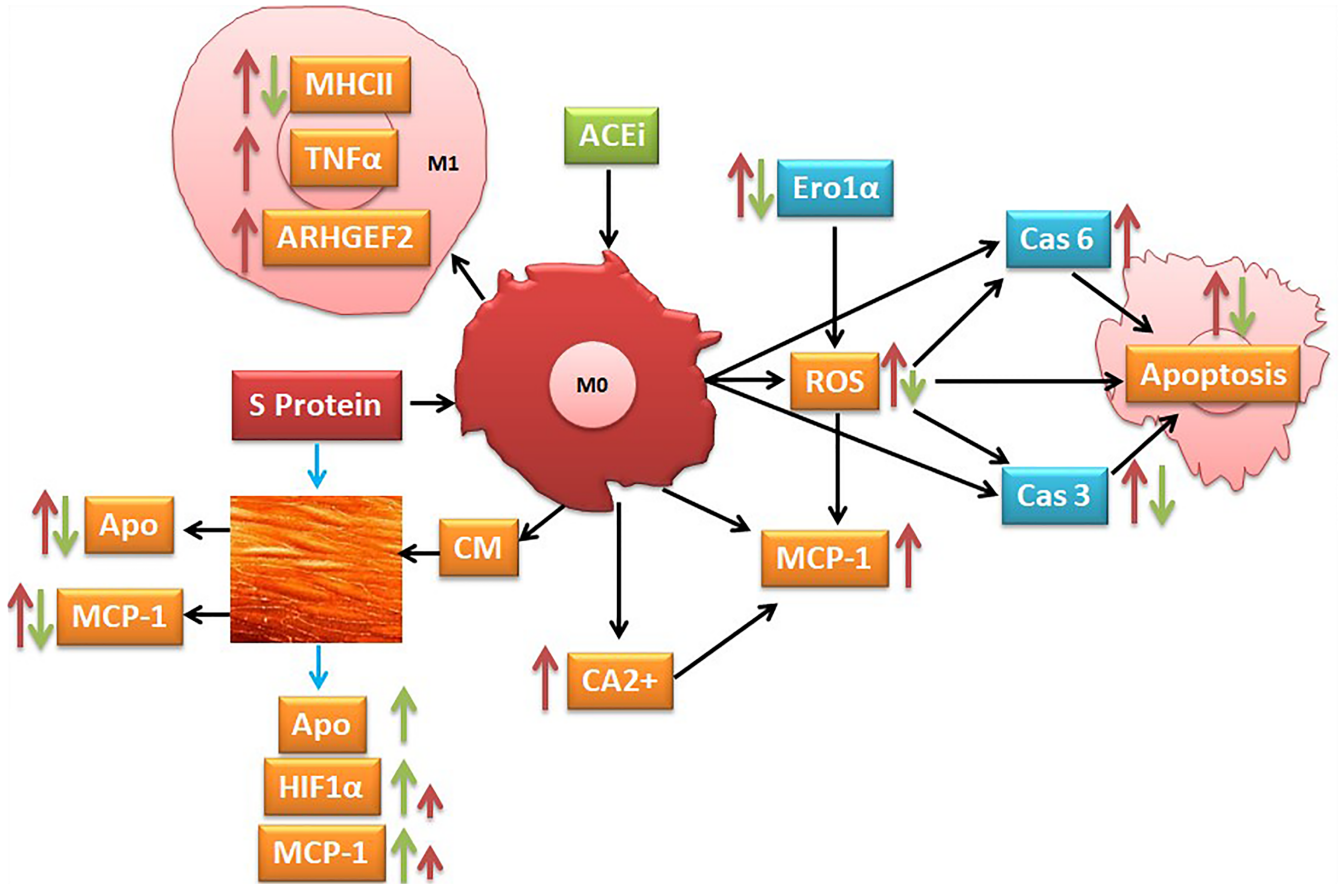


## Introduction

Severe acute respiratory syndrome-related coronavirus 2 (SARS-CoV-2), causing coronavirus disease 2019 (COVID-19), rapidly spread in 2019, creating a pandemic that severely affected health systems and the global economy and imposed a burden on governments and society (1). SARS-CoV-2 infection manifests with symptoms similar to the common cold; however, it may progress to severe disease characterized by an acute respiratory syndrome related to viral infection induced hyperinflammatory responses, usually associated with deadly hypercytokinemia and organ dysfunction (2, 3). Due to the heterogeneity of the COVID-19 clinical phenotypes and the variability of drug responses, the immune system interactions with the virus infection are mostly unclear, and the mechanisms regulating these variable outcomes are yet to be determined.

A viral infection stimulates the innate and adaptive immune systems. As a result, several cytokines and chemokines are secreted to attract specialized immune cells to the site of the infection. A disturbed immune response is a major signature of SARS-CoV-2 infection, characterized by the presence of exhaustive T cells, dysfunctional monocytes, macrophages, B cells, and natural killer (NK) cells (4). These dysregulations impair the clearance of the virus and cause hyperinflammation associated with an abnormal cytokine release, the “cytokine storm,” a precursor to cell and organ damage (5). The cytokine storm is triggered by the binding of the SARS-CoV-2 spike protein to ACE2, expressed in different tissues and organs such as the heart, kidney, pancreas, small intestine, and endothelial cells (6–8), and monocytes and macrophages (9, 10). This causes the hypersecretion of cytokines resulting in harmful damage of local tissues (11). Previously, an *in vitro* study indicated that the SARS coronavirus spike protein induced an innate immune response and suggested that the proinflammatory mediators in the monocytes and macrophages could be amplified by the S protein interactivity (12). The monocytes and macrophages play a crucial role in this process (2, 13).

Monocyte/macrophages and lymphocytes are the cornerstone of immune responses during infection. Throughout the viral infection, activated macrophages produce different chemokines such as CCL12, MCP-1, and CCL7, resulting in the recruitment and infiltration of more monocytes and macrophages to the site of inflammation, which results in a hypercytokine release, for example, interleukin (IL)-6, TNFα, and IL1-β. As a consequence, this exacerbates the disease symptoms (14), specifically the acute respiratory distress syndrome (ARDS), which is the deadliest end organ damage in COVID-19 (9). This proinflammatory macrophage microenvironment modulates the immune response in COVID-19 patients (15). An alteration of the macrophage phenotypes due to a differential gene expression is correlated with disease severity (15, 16). In addition to the cytokine release and immune cell activation, the monocyte-derived macrophages may potentially transmit the virus to peripheral lymphocytes (17), a role that is not yet fully elucidated for SARS-CoV-2 coronavirus.

The dysfunction of the adaptive and innate immune system leads to deleterious effects on blood cells and the endothelial components, resulting in the failure of the immune responses and potential injuries in systemic vasculature (18, 19). A single layer of endothelial cells (ECs) forms the endothelium and is the first line in contact with circulating lymphocytes and monocytes (20). Pulmonary EC dysfunction is a main characteristic of ARDS (21), which is a major signature of COVID-19 progression to the severe stage (22). SARS-CoV-2 infection exerts detrimental effects on the endothelium of the small vasculature by inducing endothelial inflammation, disturbing the vessel barrier integrity and contributing to the coagulation cascade (23, 24), possibly causing thrombosis (25) and endotheliitis (26).

Most of the severe COVID-19 cases are elderly patients with comorbidities, presenting frequently with cardiovascular diseases, hypertension, and diabetes, which are significantly accompanied by endothelial dysfunction and the dysregulation of the renin–angiotensin system (RAS). The majority of these patients are taking RAS inhibitor treatment, including ARBs and ACEi (27, 28).

The viral surface spike (S) protein facilitates the SARS-CoV-2 entry into the target cells *via* binding to the ACE2 receptor (29). ACE2 is a crucial component of RAS, which is expressed in several cells and organs such as macrophages and endothelial cells (30, 31). The dysregulation of the ACE2 expression or function provokes an imbalance in the RAS activities and angiotensin II pathophysiological effects, which are corrected with ARBs and ACE inhibitors (32–34). Most of the studies reported that treatment with ACE inhibitors increased ACE2 expression (35).

Spike protein binding to ACE2 is not the only mechanism required for viral entry, as the CD147 transmembrane glycoprotein may also mediate viral entry to the target cells (36). It is unclear whether there are more pathways used by the virus to enter into the target cells. During SARS-CoV-2 infection, the binding of the S protein to the surface cells may activate different pathways, rather than a simple entry to the host cell. An understanding of the virus interaction with the monocyte/macrophages and endothelial cells is crucial to understand the viral interaction with the cellular microenvironment. *In vitro* studies using S protein to mimic virus stimulation of monocyte macrophages, ECs, or peripheral blood mononuclear cells (PBMCs) can support our understanding of the viral–host cell interaction. We hypothesized that the SARS-CoV-2 spike protein (SP) may possibly activate the macrophages, HUVECs, and PBMCs, resulting in various inflammatory responses. In this study, we mechanistically provide evidence that the S protein may activate the inflammatory markers and stimulate oxidative stress in the THP-1-macrophages, PBMCs, and HUVEC cells and that an ACE inhibitor (perindopril) potentially mediates these effect.

## Materials and Methods

### Cell Lines

The THP-1 cells (ATCC, TIB-202) were cultured in Roswell Park Memorial Institute (RPMI) 1640 supplemented with 10% fetal bovine serum (Gibco), 2-mercaptoethanol of 0.05 mM, and 1% penicillin/streptomycin antibiotic (Gibco), and incubated in a humidified 37°C, 5% CO_2_ incubator. Phorbol-12-myristate 13-acetate (PMA, 10 nM) (Sigma-Aldrich) was used to differentiate the cells toward a macrophage phenotype after incubation for 72 h. The cells were washed with phosphate-buffered saline (PBS) to remove the PMA-supplemented media and rested in fresh PMA-free media for 24 h before being used for experiments. Human-umbilical vein/vascular endothelium cells (HUVECs) (ATCC, CRL-1730) were cultured in endothelial cell medium (Gibco) supplemented with 10% FBS (Gibco) and 1% penicillin/streptomycin antibiotic (Gibco), and incubated in a humidified 37°C, 5% CO_2_ incubator.

### SARS-CoV-2 S Glycoprotein Stimulation of the Cells

The SARS-CoV-2 (2019-nCoV) Spike S1 + S2 ECD-His recombinant protein was obtained from Sino Biological (40589-V08B1), reconstituted and used as recommended. The purified product from baculovirus-insect cells contained <1 endotoxin unit (EU) per microgram of S protein. The SARS-CoV-S protein was diluted in fresh RPMI 1640 medium before each experiment. The PBMCs and THP-1-differentiated cells and HUVECs were cultured in 96-well plates (100–200 × 10^3^ cells/well). Before stimulation, the cells were washed twice with PBS and treated with SARS-CoV-S protein (100 nM) or mock treated, and incubated for 24 h or 48 h in a 37°C, 5% CO_2_ incubator. To polarize the THP-1-differentiated cells to M1/M2, the cells were incubated for 24 h with IL-4 (20 ng/ml) and IL-13 (20 ng/ml) for M2-like-macrophage polarization (37) and interferon (IFN)-gamma (50 ng/ml) and lipopolysaccharide (LPS) (10 ng/ml) for M1 classical macrophage activation (38) after the stimulation with the S protein. The cells were washed twice with PBS and prepared for the experiments. The supernatant was collected as conditioned media (CM) from the S-protein stimulated THP-1-like macrophages and stored in −80°C (100 µl/aliquot) for co-culture essays with HUVECs to be used in subsequent experiments.

### Peripheral Blood Mononuclear Cells Isolation and Culture

Heparinized venous blood was collected from healthy donors. The PBMCs were isolated using Ficoll–Paque density gradient centrifugation. The PBMCs were seeded in a 96-well plate, approximately at 3 × 10^5^ cells in 100 μl of RPMI per well. For activation, the cells were stimulated with anti-CD3 (OKT3, eBioscience, 10 μg/ml) and anti-CD28 (CD28.2, eBioscience, 10 μg/ml) and treated with SARS-CoV-2 (2019-nCoV) Spike protein (100 nM, Sino Biological) for 24 h. To measure the intracellular cytokine release, the cells were stimulated with PMA (50 ng/ml, Sigma-Aldrich) and ionomycin (1 μg/ml, Sigma-Aldrich) for 4–6 h in the presence of monensin (1 μg/ml, Sigma-Aldrich), collected and analyzed by flow cytometry.

### Flow Cytometry

The flow cytometric analysis was performed to evaluate the effect of the different treatments on the cell phenotype, inflammatory markers, and apoptosis as previously described (39). At the end of each experiment, the cells were washed twice and incubated for 15 min with PBS supplemented with 1% bovine serum albumin (BSA) to prevent non-specific binding of antibodies and conjugated with antibodies for TNFα, MHC-II, HLA-DR, CD14, CD206, MCP-1, HIF1α, CD4, CD8, CD56, IFNγ, and IL-17 (**Supplementary Table S1**) for the analysis of different populations (**Supplementary Figures S8, S9**). For apoptosis, the cells were harvested and resuspended in 400 μl binding buffer before adding 5 μl Annexin V-FITC and incubated at 4 °C for 30 min in the dark. After that, the cells were analyzed immediately using BD FACSCanto II and FACSDiva Software (BD Bioscience). Supplementary experiments were performed using LSR Fortessa (BD Bioscience).

### Wound Healing

The HUVECs were seeded on a 96-well plate and grown to 90–100% confluency, then wounded using a 10-μl sterile pipette tip applied perpendicularly to the well diameter in a straight line. The cells were washed twice with serum-free medium before their incubation with different treatments. The images were captured every 6 h for 48 h using an EVOS FL Auto Microscope (Thermo Fisher Scientific). Finally, the remaining wound area was measured using ImageJ software and analyzed. The cells were maintained in a humified chamber with 5% CO2 at 37°C throughout the protocol.

### Mass Spectrometry Analysis

#### Proteomics Sample Preparation

The cells pellets were lysed in radioimmunoprecipitation assay (RIPA) buffer (Thermo Fisher Scientific) followed by a total protein measurement using the Pierce™ BCA Protein Assay Kit (Thermo Fisher Scientific). For each sample, 100 µg of the proteins was precipitated using methanol/chloroform/water. The proteins pellets were dissolved in 8 M urea/500 mM Tris–HCl buffer and reduced with 500 mM Tris (2-carboxyethyl) phosphine hydrochloride, followed by alkylation with 500 mM 2-chloroacetamide. The proteins were digested into peptides using trypsin with a ratio of 1:50 (protein to trypsin) and incubated at 37°C overnight. Prior to the mass spectrometry analysis, the peptides were desalted and cleaned up using the Pierce™ Peptide Desalting Spin Columns, according to the manufacturer’s protocol (Thermo Fisher Scientific).

#### Nano-LC-MS/MS Analysis

The analysis of all the samples was performed on an Ekspert nanoLC 425 (Eksigent, Dublin, CA, USA) coupled to a TripleTOF 5600 (Sciex, Concord, Canada). The peptides were trapped on C18, and separation was achieved by reverse phase chromatography, using a C18 nano-LC column (3C18-CL, 75 µm × 15 cm, Eksigent, USA) with the following elution gradient: 0–1 min, 5% B; 1–60 min, 5–30% B; 60–66 min, 30–80% B; 66–74 min, 80% B; 74–85 min, 80–5% B; and 85–100 min, 5% B using mobile phase A (0.1% HCOOH in water) and mobile phase B (0.1% HCOOH in Acetonitrile). The MS acquisition method was operated in data-dependent acquisition (DDA) mode with the following criteria: the MS1 range was set at 400–2,000 *m*/*z* and the 10 most intense precursor ions with charge states (+2 to +5), exceeding 200 cps, were selected for fragmentation. The MS2 range was set at 400–1250 *m*/*z*, and the fragmentation was performed using Rolling collision energy with a collision energy spread (CES) = 5 eV. The instrumental parameters for MS1 and MS2 were set as GS1 = 25, GS2 = 0, CUR = 25, ISVF = 2250, and IHT = 60.

#### Protein Data Processing

The protein identification was performed using ProteinPilot Software (v 4.5, Sciex). The MS/MS data files were searched against the Uniprot Proteome Humans database (downloaded on October 29, 2019). The detected protein threshold was set with 0.05 unused protscore confidences, and the false discovery rate (FDR) analysis was checked. The protein quantification was performed using Skyline software (v 20.1.0.31). The mass spectrometry proteomics data have been deposited in the ProteomeXchange Consortium *via* the PRIDE (40) partner repository with the dataset identifier PXD025516. Tableau software was used for the volcano plot visualization. A GO enrichment analysis and pathway analysis were performed using The Database for Annotation, Visualization and Integrated Discovery (DAVID) and STRING.

### MTT Essay

To assess the cytotoxicity of the S protein on HUVEC cells, a 3-(4, 5-dimethylthiazol-2-yl)-2,5-diphenyltetrazolium bromide (MTT) assay was performed using 5 mg/ml MTT reagent and 8 × 10^4^ cells per well in a 96-well plate. After 2–3 h incubation at 37 °C, 200 µl of DMSO (Sigma Aldrich, USA) was added, and formazan was quantified using a spectrophotometer (Molecular Devices, USA) to read the absorbance at 570. The percentage cell viability was analyzed using the formula: % viable cell = (mean of OD_test_/mean of OD_control_) × 100.

### Measurement of the ROS Levels

For the reactive oxygen species detection, the CellROX™ (ThermoFisher scientific) Deep Red fluorogenic probe was used. ROS production was measured using Flow cytometric analysis as previously described (41, 42) with modifications. Briefly, cells were incubated with 4 μM CellROX for 25 min at 37°C in the dark. The ROS levels were measured using a BD FACSCanto II flow cytometer; the fluorescence was captured at 644/665 nm of excitation/emission light and the values reported as percentage of positive cells.

### Determination of the Intracellular Calcium

The intracellular calcium measurement was performed with Fluo4/AM fluorescence (Biotium, Fremont, CA, USA). After binding to Ca_2_^+^, the Fluo4 increase fluorescence as an AM ester is hydrolyzed intracellularly by esterases. The cells were incubated with CaCl_2_ buffer (5 mM) for 15 min, and 5 μM of Fluo4/AM was added and incubated for 30 min at 37°C in the dark. The cells were washed twice, resuspended in 400 µl of 5 mM CaCl_2_ buffer, and analyzed by flow cytometer at 488/530 nm excitation/emission wavelengths.

### Statistical Analysis

Prism^®^ 5.0 (GraphPad Prism Software Inc., La Jolla, CA, USA) software was used to analyze the data, which are presented as mean ± SEM. A Student’s t-test was used to assess the statistical significance between two groups, and a one-way analysis of variance (ANOVA) compared more than two groups.

## Results

### The Effects of the Spike (S) Protein on Cell Viability, the Inflammatory Markers, and the Polarization of THP-1-Like Macrophages

**Figure 1:**
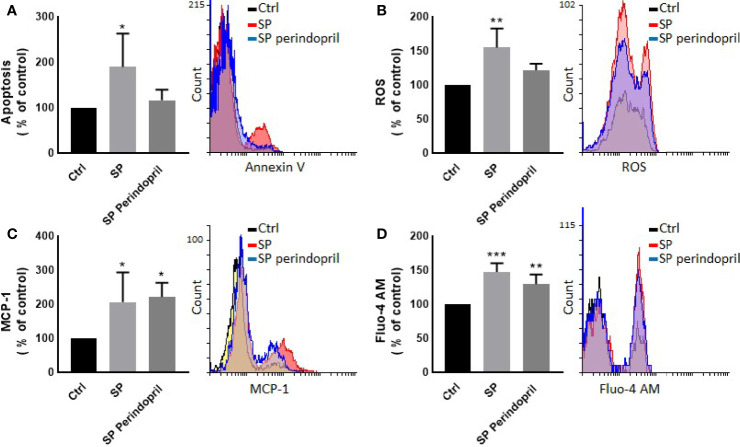
Effects of spike (S) protein on cell viability and inflammatory markers. Thp-1 macrophages were mock treated (Ctrl), stimulated with S protein (SP) (100 nM) or SP with ACE inhibitor (SP Perindopril, 100 µM), perindopril, added 2 h before SP. **(A)** Apoptosis was measured by Annexin V binding essay. **(B)** Oxidative stress was evaluated by reactive oxygen species (ROS) expression. **(C)** Monocyte chemoattractant protein-1 (MCP-1) expression essay. **(D)** Fluo4 fluorescence as a function of cytosolic free Ca^2+^ intracellular calcium was performed using flow cytometry analysis. The data are presented as mean ± SEM, n = 4–5. Ordinary one-way ANOVA followed by Dunnett’s test: *p < 0.05, **p < 0.01,***p < 0.001.

**Figure 2:**
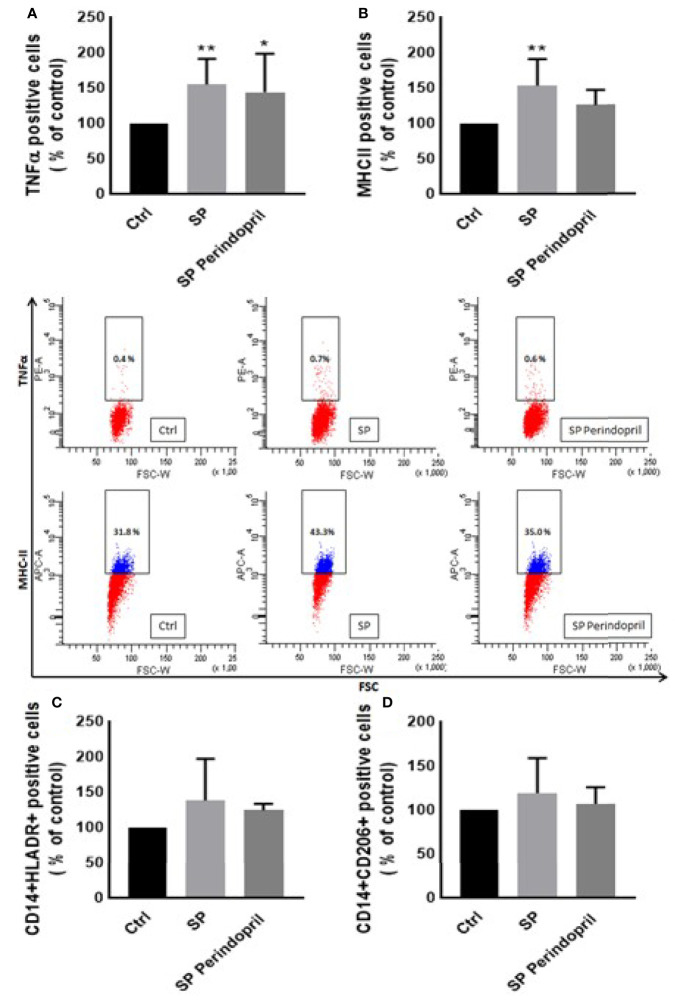
Effects of spike (S) protein on cell polarization of THP-1-like macrophages. Thp-1 macrophages were mock treated (Ctrl), stimulated with S protein (SP) (100 µM) or SP with ACE inhibitor (SP Perindopril, 100 µM), perindopril, added 2 h before SP. After cell differentiation, the effect of SP stimulation on **(A)** TNFα, **(B)** MHCII, **(C)** HLA-DR, and **(D)** CD206 expression was measured by flow cytometry. The data are presented as mean ± SEM, n = 4–5. Ordinary one-way ANOVA followed by Dunnett’s test *p < 0.05, **p < 0.01.

We treated the THP-1 cells with S recombinant protein containing the S1 and S2 subunits of the type I transmembrane spike protein. The receptor binding domain (RBD) is contained mainly in the S1 subunit and facilitates cell surface receptor binding. Following the exposure of the differentiated THP-1-like macrophage cells to 100 nM of S protein for 24 h, apoptosis was significantly increased compared to the control (**Figure 1A**). The activity of the cells was clearly dysregulated by a noticeable increase in the ROS levels (**Figure 1B**) and intracellular calcium release (**Figure 1D**). We were interested to observe whether the treatment with an ACE inhibitor (perindopril) (43) can mediate these effects. The effects on apoptosis, ROS, and intracellular calcium were slightly decreased after the treatment with perindopril without reaching statistical significance, except for the intracellular calcium (**Figures 1A, B, D** **)**. The monocyte chemoattractant protein 1 (MCP-1), as a major chemokine responsible for the recruitment of T lymphocytes and natural killer (NK) cells to the site of inflammation (44), was highly increased after SP treatment with no reduction after treatment with perindopril (**Figure 1C**). The THP-1-like macrophage cells are highly flexible towards their differentiated phenotype. Depending on the type of stimulus and the cytokines released within the inflammatory microenvironment conditions, they can polarize to a proinflammatory (M1) or anti-inflammatory (M2)-like phenotype. The S glycoprotein stimulation significantly increased the M1-like phenotype markers, including TNFα (**Figure 2A**), MHCII (**Figure 2B**), and HLA-DR (**Figure 2C**). However, there was no significant effect on the M2-like phenotype marker or the mannose receptor expression (**Figure 2D**). Treatment with perindopril slightly decreased the MHCII expression with no significant effect on the other markers.

### Effects of the Spike (S) Protein on the Immune Response of the Peripheral Blood Mononuclear Cells

**Figure 3:**
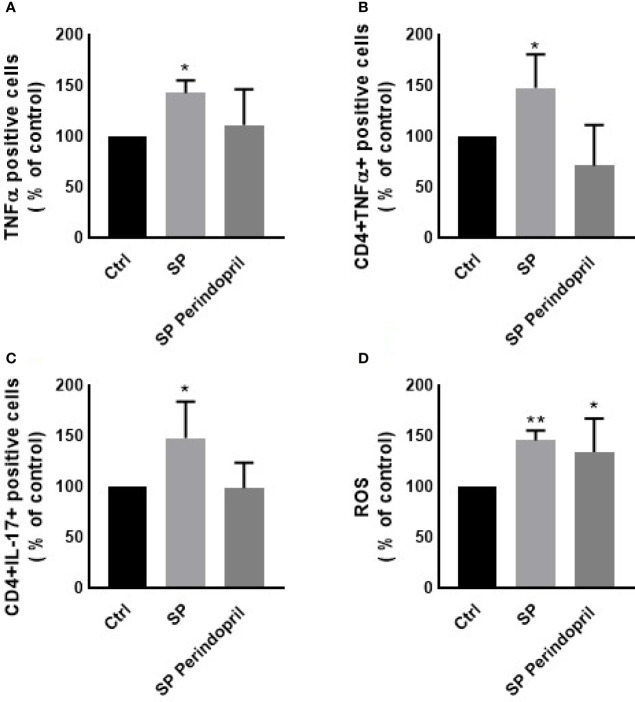
Effects of spike (S) protein stimulation on PBMCs. PBMCs were mock treated controls (Ctrl), stimulated with S protein (SP) (100 µM), or SP with ACE inhibitor (SP Perindopril, 100 µM), perindopril, added 2 h before SP. Flow cytometry analysis was performed for measurement of **(A)** expression of TNFα in PBMCs, **(B)** expression of TNFα on CD4 positive T cells, **(C)** expression of intracellular IL-17 on CD4 T cells, and **(D)** ROS production on PBMCs. The data are presented as mean ± SEM, n = 4. Ordinary one-way ANOVA followed by Dunnett’s test: *p < 0.05, **p < 0.01.

To determine the effect of the S protein treatment on the intracellular production of the cytokines INFγ, TNFα, and IL-17, PBMCs were treated with S protein at 100 nM or in combination with perindopril (100 µM) for 24 h and stimulated with PMA (50 ng/ml) plus ionomycin (1 μg/ml) for 5 h. After the mitogenic stimulation, the S-protein-treated PBMCs produced significantly higher levels of TNFα (**Figure 3A**). Similarly, this was observed in the isolated CD4 T cells compared with the mock-treated controls (**Figure 3B**). The IL-17 expression was upregulated in the CD4 helper T cells after S protein stimulation compared to the mock-treated controls (**Figure 3C**). However, we did not observe significant effects on IFNγ production in the PBMCs, T helper cells, cytotoxic T cells, or NK cells (**Supplementary Figure S3A–C**). The combination of S protein stimulation with perindopril treatment decreased the TNFα in the PBMCs, IL-17, and the TNFα expression in CD4 cells (**Figures 3A–C**).

It is known that ROS play crucial roles in the physiological processes in cells. The dysregulation of the redox balance increase free radicals, which cause damage and alters several cellular functions (45, 46). During the ARDS phase of the infection in COVID-19, ROS induced cell damage and altered signaling pathways (45). The exposure of the PBMCs to the S protein for 24 h significantly increased the ROS levels compared to the mock-treated controls. The treatment of perindopril did not change this effect (**Figure 3D**).

### Effects of the Spike Protein on the HUVEC Cells

**Figure 4:**
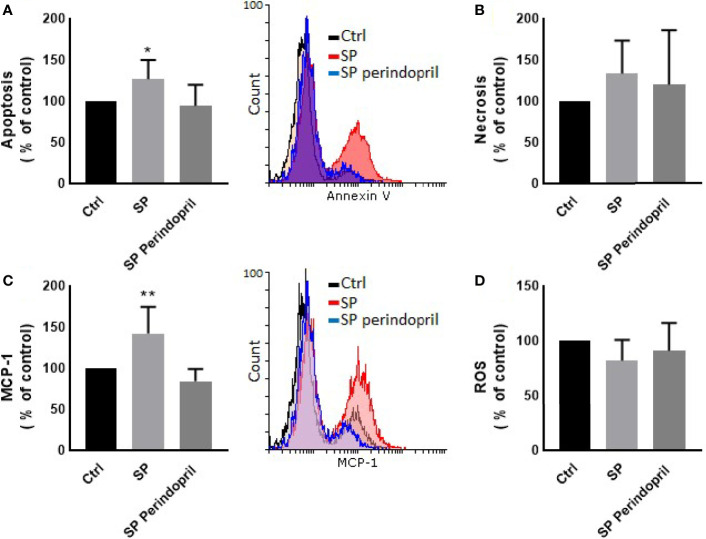
Effects of conditioned media of Thp-1 macrophages cells stimulated with spike (S) protein on HUVEC cells. Thp-1 macrophages were mock treated (Ctrl), stimulated with S protein (SP) (100 µM), or SP with ACE inhibitor (SP Perindopril, 100 µM), perindopril, added 2 h before SP. After 24 h incubation, conditioned media (CM) was collected and used for HUVEC cell culture. **(A)** Apoptosis was measured by Annexin V binding essay. **(B)** Necrosis detection was evaluated by internalization of propidium iodide (PI). **(C)** Monocyte chemoattractant protein-1 (MCP-1) expression. **(D)** ROS detection was measured by flow cytometry. The data are presented as mean ± SEM, n = 4. Ordinary one-way ANOVA followed by Dunnett’s test: *p < 0.05, **p < 0.01.

**Figure 5:**
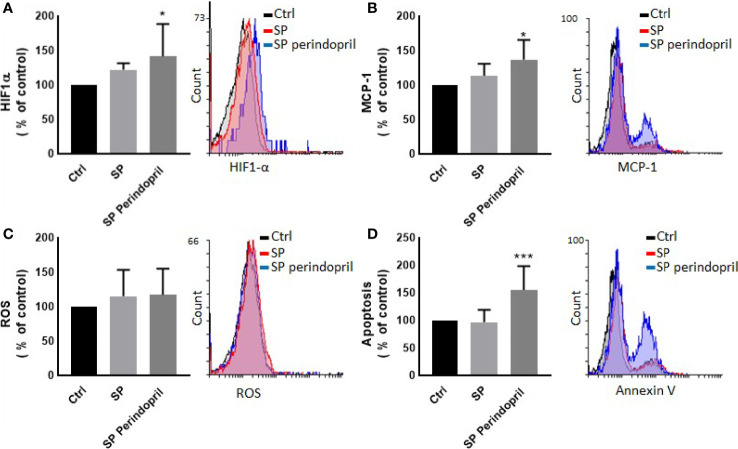
Effect of spike (S) protein stimulation on HUVEC cells. HUVEC cells were mock treated (Ctrl), stimulated with S protein (SP) (100 µM) or SP with ACE inhibitor (SP Perindopril, 100 µM), perindopril, added 2 h before SP. **(A)** Hypoxia-inducible factor 1-alpha (HIF1α) expression. **(B)** Monocyte chemoattractant protein-1 (MCP-1) expression. **(C)** ROS detection. **(D)** Apoptosis evaluated by Annexin V binding essay was measured by flow cytometry. The data are presented as mean ± SEM, n = 4–5. Ordinary one-way ANOVA followed by Dunnett’s test *p < 0.05, ***p < 0.001.

To determine the effect of the S protein on endothelial cells, the HUVEC cells were incubated with CM from THP-1-like macrophage cells, stimulated with 100 nM of S protein or in combination with perindopril (100 µM) for 24 h. The CM from the S-protein-stimulated THP-1 macrophage cells increased apoptosis (**Figure 4A**) and MCP-1 expression (**Figure 4C**) without significant change in necrosis (**Figure 4B**). This effect was reduced when the THP-1 macrophage cells were incubated with a combination of S protein and perindopril (**Figures 4A, C** **)**. However, there was a non-significant effect on ROS generation (**Figure 4D**). We evaluated the effect of the direct stimulation of the HUVEC cells with S protein. The results show no effect on the MCP-1 (**Figure 5B**), apoptosis (**Figure 5C**), or ROS (**Figure 5D**). Interestingly, the combination of the S protein with an ACE inhibitor increased all the previous effects. We also tested the effect on hypoxia-inducible factor 1-alpha (HIF1α), another marker of endothelial cell damage (**Figure 5A**). We observed a tendency to increase after the stimulation with the S protein that was significantly increased in the combination with perindopril (**Figure 5D**).

### Wound Assay

To evaluate the effect of the S protein on HUVEC migration, the wound healing essay was performed for 48 h. The percentage wound healing was performed continuously, every 6 h of the 48 h of incubation. The general spectrum was almost similar in all groups; however, we have to mention that the trend of wound closure slightly fluctuated at 24, 36, and 48 h in both the S-protein-stimulated groups, compared to the controls, without any statistical significance (**Supplementary Figure S4**).

### Differential Effects on Protein Expression in THP-1-Like Macrophages

**Figure 6:**
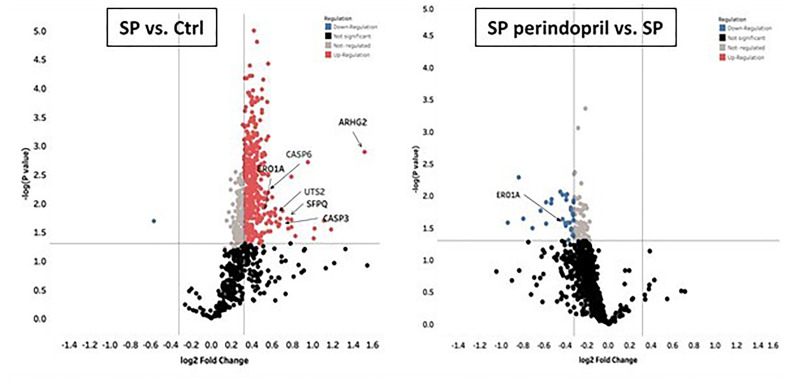
Mass spectrometry analysis of THP-1 macrophages cells stimulated with spike (S) protein. Volcano plot display differentially expressed proteins in cells stimulated with S protein (SP) (100 µM) versus mock treated (Ctrl) or in presence of ACE inhibitor (SP Perindopril, 100 µM): colored circles are significant proteins (p < 0.05), red is upregulated proteins with fold change ≥1.25, and blue is downregulated protein with fold change ≤0.83. Student’s t-test used for statistical analysis. Gray circles are non-regulated proteins, and black are proteins with non-significant p-values.

To evaluate the effect of the S protein on the protein expression profile of the monocyte-like macrophages, a label-free quantitative proteomics analysis was performed. The statistical criteria for the differentially expressed protein analysis was set with a fold change ≥1.25 for upregulation and ≤0.83 for downregulation, with a *p* < 0.05. The data indicated the upregulation of 387 proteins in the S-protein-treated cells compared to the mock-treated cells and 56 downregulated proteins in the cells treated with S protein and perindopril (100 µM), compared to S protein only (**Supplementary Table S2**). We focused mainly on caspase-6, caspase-3, ER, oxidoreductin-1 alpha (Ero1α), and Rho guanine nucleotide exchange factor 2 (ARHGEF2) proteins that were upregulated in the S protein group and decreased or blunted in the S protein plus perindopril (100 µM) group, as these are highly involved in apoptosis, ROS generation, and macrophage polarization (**Figure 6**).

All the significant differentially expressed proteins were searched against the DAVID program to identify the enriched biological terms, including the biological processes, molecular function, and cellular component (47). We considered only the significant and most enriched terms in all the three pairwise comparisons. Through the GO enrichment analysis of differentially expressed proteins in SP compared to control or SP plus perindopril groups, we found that the most enriched terms were protein binding, cell–cell adhesion, and cytosol in molecular function, biological processes, and cellular component, shown in **Supplementary Figures S5, S6**. The pathway enrichment analysis was conducted by STRING, on the basis of Reactome database (48, 49).

The most significant enriched pathway associated with the stimulation of spike protein in the THP1-macrophage cells was apoptosis, displayed in **Supplementary Figure S7A**. The pathway enrichment analysis was also performed for SP + Perindopril vs. SP (**Supplementary Figure S7B**). All other results are reported in **Supplementary Table S2**. However, further experiments and an increased number of samples may be of interest to identify potentially new pathways.

## Discussion

The literature reports that the monocyte/macrophage expression and function in SARS-CoV-2-infected patients are disturbed and that the macrophages highly expressed the ACE2 receptors, mostly in lung tissues (50–52). In agreement with these observations, we indicated that the SARS-CoV-2 S protein stimulation activates THP-1-like macrophage *via* an increase in ROS and intracellular calcium release, which is likely the cause of the significant increase in cell apoptosis compared to the mock-treated cells. The S-protein-induced apoptosis may be due to the caspase pathway activation, confirmed by the proteomic analysis on the THP-1-like macrophages; we observed that caspase-6 and caspase-3 were upregulated in cells stimulated with S protein, suggesting their role in the execution of the apoptotic cascade (Graphical Abstract). Notably, only caspase-3 was downregulated in the cells treated with S protein and ACEi compared to the S protein alone, which may explain the decrease in apoptosis in the cells stimulated with S protein and treated with perindopril, mostly *via* the caspase-3 pathway.

Apoptosis of lung endothelial and epithelial cells ultimately trigger vascular and alveolar damage. This process is modulated by proinflammatory mediators, such as TNFα, CCL3, and CCL2 (MCP-1), leading to hypoxia, ARDS, and possibly death for COVID-19 patients (2). MCP-1 is a crucial chemokine responsible for NK and T-cell recruitment (53, 54). Our results showed that S protein stimulation for 24 h significantly increases MCP-1 expression in THP-1-like macrophages, which may be triggered by ROS generation, as ROS-mediated signaling pathways in angiotensin II-induced MCP-1 expression were reported (55). In our study, ROS was significantly upregulated in cells treated with S protein; however, the combination with perindopril blunted this upregulation without a significant effect on MCP-1, which may be due to other pathways.

During viral infection, the phagocytosis of viruses by macrophages triggers an immune response and assists in tissue regeneration and homeostasis (56). Such activity is usually modulated by the inflammatory microenvironment and cytokines that polarize the THP-1-like macrophage toward a classically M1-like phenotype, or alternatively M2-like phenotype (57), with different functions and cytokine profile release. We showed that the treatment of the THP-1-like macrophage with spike protein increased the M1-like phenotype markers, TNFα, and MHCII, increasing HLA-DR, which was not statistically significant. However, our results did not show any significance in the CD206 M2-like phenotype marker and no effect of the pretreatment with an ACE inhibitor. The RhoA function depends on the family of guanine-nucleotide exchange factors (GEFs). Rho GEFs inhibition and RhoA deletion affects the organization of the Golgi complex, crucial for normal macrophage functions such as phagocytosis. It was reported that the GEFs are crucial for the Ras homolog family (RhoA) and that the inhibition of GEFs induces macrophage dysfunction (58). We found that the Rho guanine nucleotide exchange factor 2 (ARHGEF2) was significantly upregulated in the S protein cells, which may explain the polarization of the THP-1-like macrophages toward a more inflammatory like phenotype, validated by the M1-like macrophage markers, such as TNFα, MHCII, ROS, and MCP-1 (Graphical Abstract).

To further analyze the effect of the released cytokines and proteins, we cultured endothelial cells with collected CM from the THP-1-like macrophage treated with S protein. We found that the HUVEC cells cultured with CM from cells treated with S protein, expressed more MCP-1 compared to the CM from the mock control cells. Interestingly, the MCP-1 level decreased significantly in the HUVECs cultured with CM from the macrophages pretreated with perindopril. However, and as we presented previously, the inhibition of ACE in the THP-1-like macrophage treated with S protein does not change the MCP-1 expression, which may be explained by a different local activation of the RAS pathways. CM from macrophages treated with SP include all released components. However, potential effects of remnant S protein should not be neglected. To evaluate the direct effect on the endothelial cells, HUVECs were treated for 24 h with S protein, and the inflammatory markers were determined using flow cytometry. The MCP-1 expression does not change following the treatment with S protein, although it was exaggerated in the cells pretreated with perindopril, possibly due to upregulation of ACE2 expression and S protein binding. The same effect was observed in the HIF-1alpha expression, which may be the trigger for MCP-1 upregulation, as it was reported that MCP-1 is a HIF-1α target gene and that HIF-1α is associated with MCP-1 transcription (59). No effect on ROS generation was detected in HUVECs treated directly with S protein or CM, contrary to the THP-1-like macrophages, suggesting cell specific responses to S protein stimulation.

IFNγ and TNFα are crucial cytokines involved in innate and adaptive immunity, and their dysfunction or abnormal release is a key modulator for macrophage and immune cell activation and viral clearance. To evaluate the potential immune response of lymphocytes, S protein treatment before mitogen stimulation induced the upregulation of the intracellular TNFα and IL-17 in whole PBMCs and CD4 T helper cells. This effect is possibly due to the activation of monocytes that sequentially activate T-cell subpopulations differentially, as we did not find any effect on the CD8 cytotoxic T cells. We also did not find a significant effect on IFNγ expression on the CD4, CD8, or NK cells, suggesting that the S protein binding to ACE2 may not be a stimulator for any pathway related to IFNγ release following mitogen activation.

The abnormal generation of ROS and antioxidants are major effectors in viral replication and pathology related to viral infection (60). It was reported that the upregulation of ROS play a crucial role in the pathogenesis of different respiratory diseases such as SARS-CoV infection and that an imbalance in oxidant/antioxidant homeostasis is associated with the severity and progression of the disease (61). Cellular changes in ROS are a hallmark and trigger of death, proliferation, differentiation, metabolism, and signaling (62). We observed that the treatment of the PBMCs with S protein induces significant ROS generation compared to mock-treated cells, an effect that was slightly decreased when the cells are pretreated with perindopril. ROS dysregulation in response to S protein stimulation was observed in the THP-1-like macrophage cells and the PBMCs; however, we did not find any variation in the HUVEC cells treated with CM or directly with S protein, suggesting a differential response of cells depending on the type and function.

To highlight the role of the monocyte macrophages in the oxidative stress process, we performed a proteomic analysis, and our results show a significant increase in ER oxidoreductin-1 alpha (Ero1α) in the S-protein-treated cells compared to the mock-treated controls. The same protein was significantly decreased compared to cells treated with perindopril and stimulated with S protein, suggesting a potential pathway implicated in the ROS upregulation by S protein stimulation and angiotensin-converting enzyme inhibition following perindopril treatment, as it has been reported that the Ero1α protein is an inducer of ROS generation (63).

Collectively, our findings highlighted the evidence that S glycoprotein *in vitro* stimulation potentially promote the differential activation of THP-1-like macrophages, HUVECs, and PBMCs. S protein induces ROS generation in PBMCs and THP-1-like macrophages, most probably due to the Ero1α protein upregulation. S protein induction polarizes the THP-1-like macrophages toward the M1-like phenotype and modulates intracellular TNFα and IL-17 expression in the activated PBMCs and HIF1α and MCP-1 in HUVEC cells. These effects were blunted partially after treating the cells with an ACE inhibitor. These results indicated that the proinflammatory mediators could be stimulated by S protein interaction with immune and endothelial cells and that the SARS-CoV-2 virus infection may differentially activate host cells independently, which potentially explain the variety of clinical phenotypes and severity progress of COVID-19. In view of the fact that our study was *in vitro* using spike protein, more investigations using live virus or an animal model are highly valuable for targeting this area that may be of interest for the diagnosis and treatment of COVID-19.

## Data Availability Statement

The datasets presented in this study can be found in online repositories. The names of the repository/repositories and accession number(s) can be found below: https://www.ebi.ac.uk/pride/archive/, PXD025516.

## Author Contributions

The experiments were conducted by TB and MB. The results were analyzed by TB, BG, FA, HS, FM, HA, MA, and BG. HS was in charge of the proteomics analysis. FM was in charge of cell culture and sample preparation. TB wrote the paper, which was critically revised by all authors. All authors contributed to the article and approved the submitted version.

## Funding

This research was supported by King Abdullah International Medical Research Centre (KAIMRC) and funded by grants RC20/153/R, 2020 and RC18/171/R.

## Conflict of Interest

The authors declare that the research was conducted in the absence of any commercial or financial relationships that could be construed as a potential conflict of interest.

## Publisher’s Note

All claims expressed in this article are solely those of the authors and do not necessarily represent those of their affiliated organizations, or those of the publisher, the editors and the reviewers. Any product that may be evaluated in this article, or claim that may be made by its manufacturer, is not guaranteed or endorsed by the publisher.

## Abbreviations

S protein, spike protein; TNFα, tumor necrosis factor alpha; MHC-II, major histocompatibility complex class II; HLA-DR, human leukocyte antigen—DR isotype; NK, natural killer cells; MCP-1, monocyte chemoattractant protein 1; ROS, reactive oxygen species; HUVECs, human-umbilical vein/vascular endothelium cells; ACEi, angiotensin-converting enzyme inhibitor; RAS, renin angiotensin system; HIF1α, hypoxia-inducible factor 1-alpha; ROS, reactive oxygen species.
