# Supplementary material for: SARS-CoV-2 Coronavirus Spike Protein-Induced Apoptosis, Inflammatory, and Oxidative Stress Responses in THP-1-Like-Macrophages: Potential Role of Angiotensin-Converting Enzyme Inhibitor (Perindopril)
Source: Front Immunol. 2021 Sep 20;12:728896. doi: 10.3389/fimmu.2021.728896 (PMC8488399; doi:10.3389/fimmu.2021.728896)
Supplement: Supplementary file 1 [file DataSheet_1.pdf]

SUPPLEMENTAL MATERIAL

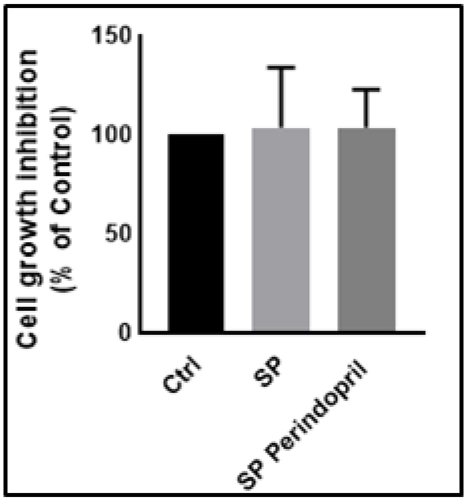

Figure S1. Cell viability determination via MTT assay. HUVECs were mock treated (Control), stimulated with S protein (SP) (100 nM) or SP with ACE inhibitor (SP Perindopril, 100 µM). Data are presented as mean ± SEM, n=3-4.

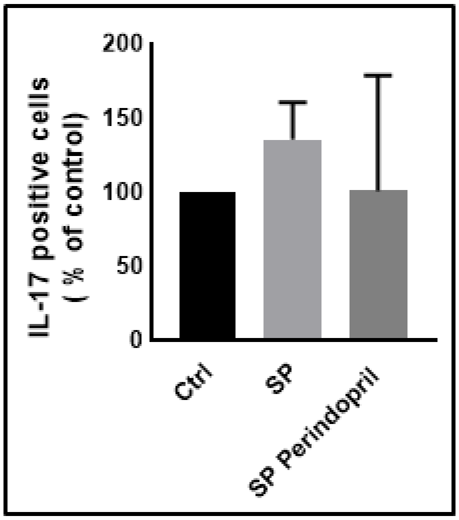

Figure S2. PBMCs were mock treated (Ctrl), stimulated with S protein (SP) (100 nM) or SP with ACE inhibitor (SP Perindropil, 100 µM). Expression of intracellular IL-17 using flow cytometry analysis. Data are presented as mean ± SEM, n=4.

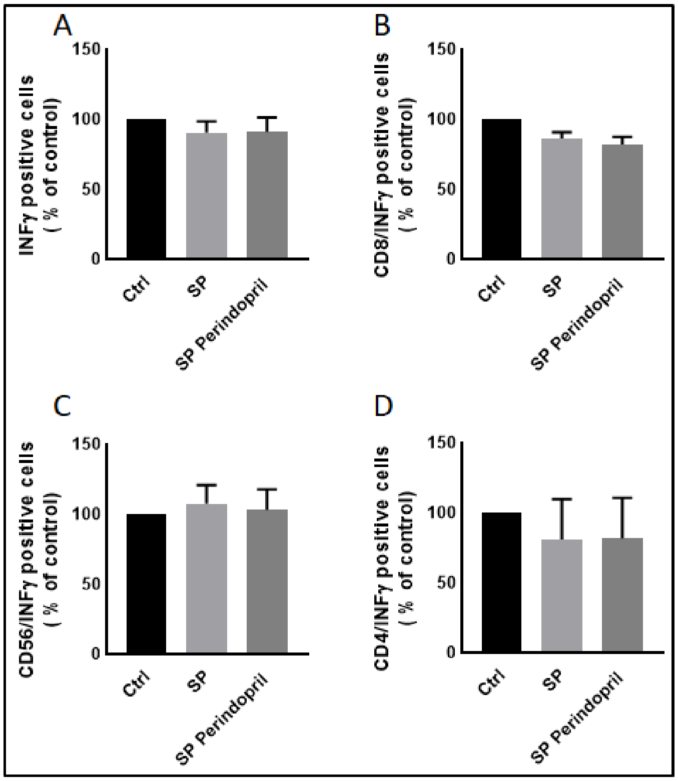

Figure S3. PBMCs were mock treated (Ctrl), stimulated with S protein (SP) (100 nM) or SP with ACE inhibitor (SP Perindopril, 100 µM). Expression of INFγ. **A)** Total PBMCs. **B)** CD8 cytotoxic T cells. **C)** CD56 NK cells. **D)** CD4 T helper cells using flow cytometry analysis. Data are presented as mean ± SEM, n=5.

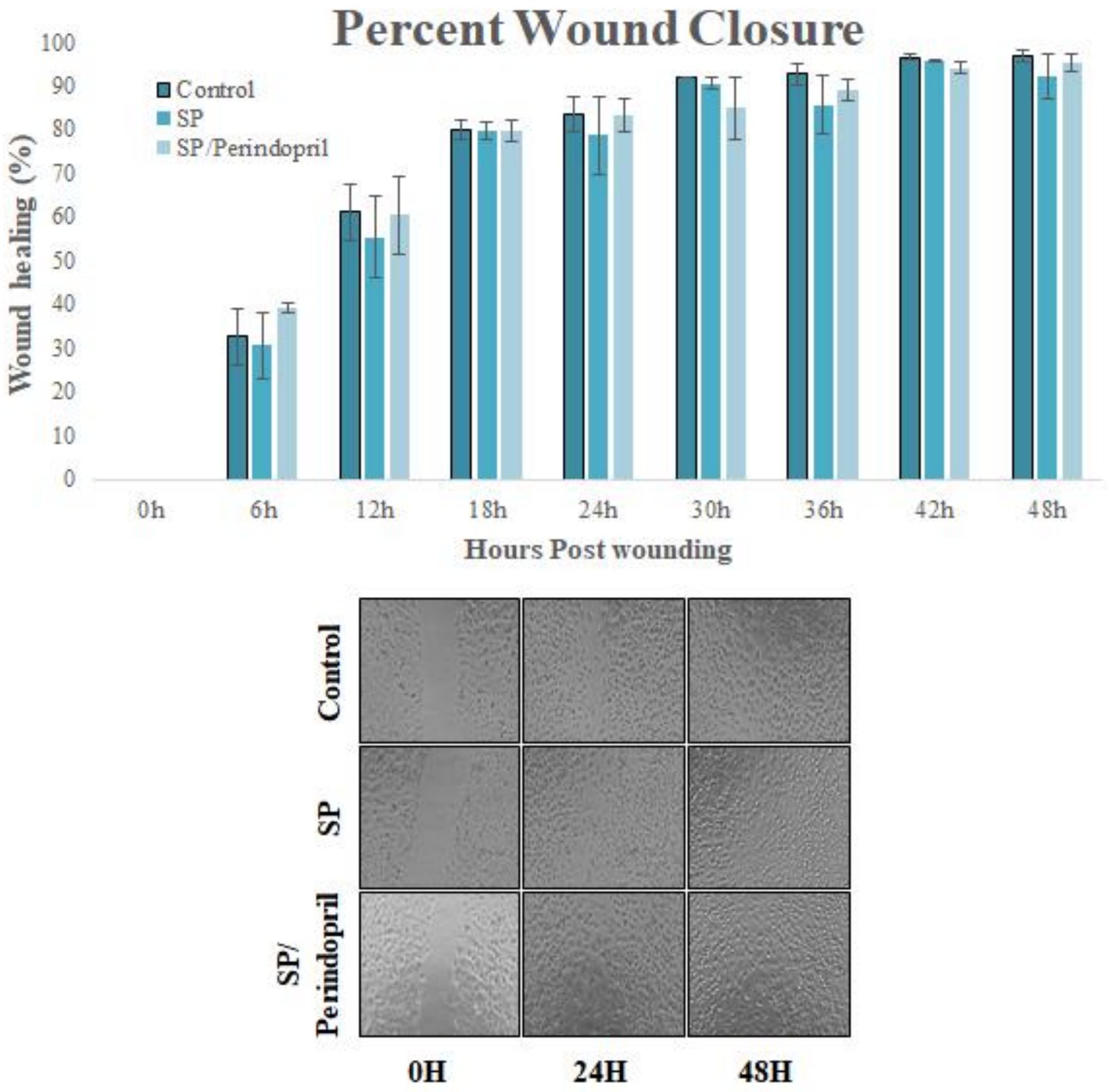

Figure S4. HUVECs were mock treated (Control), stimulated with S protein (SP) (100 nM) or SP with ACE inhibitor (SP Perindropil, 100 µM). Wound healing essay was performed during 48h. Images were captured every 6 hours for 48 hours using EVOS FL Auto Microscope. Representative images at 0H, 24H and 48H are presented. Data are presented as mean ± SEM, n=3-4.

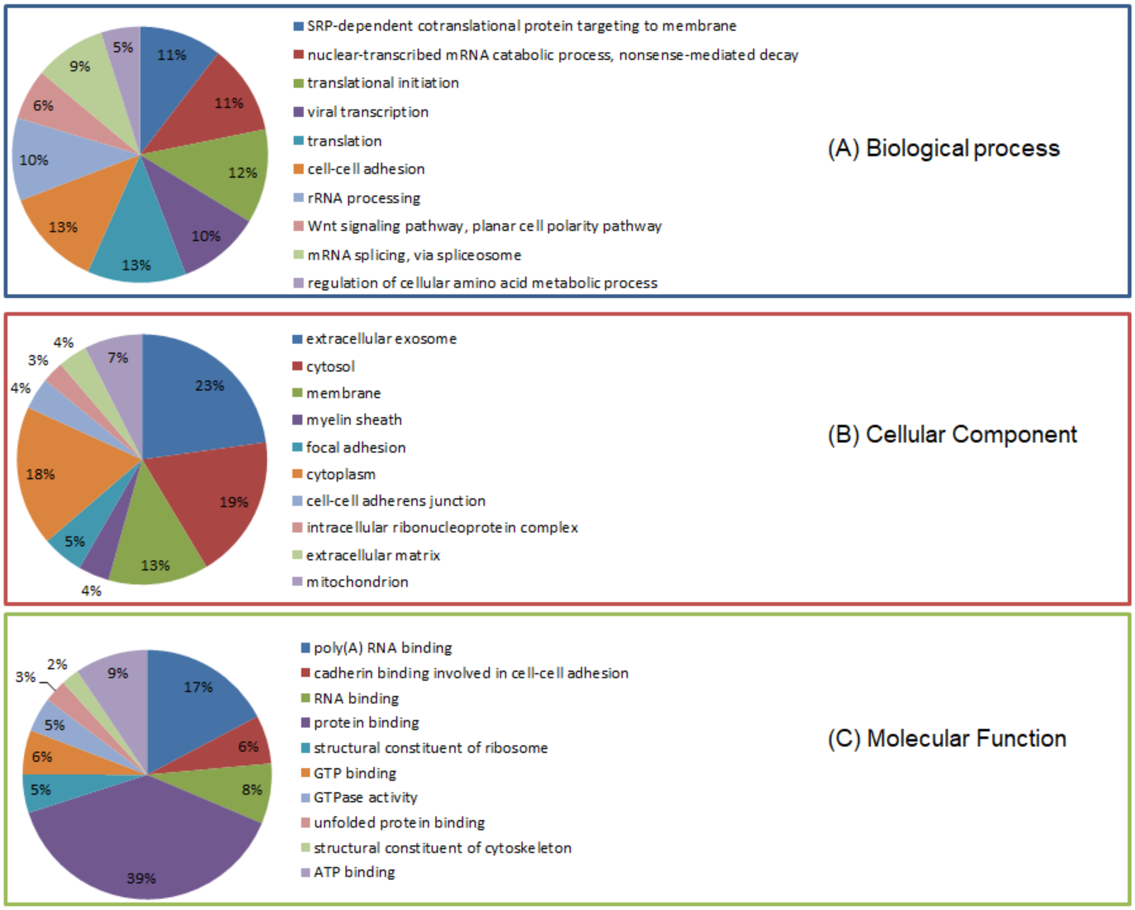

Figure S5. GO enrichment analysis of differentially expressed proteins (>1.25 fold or < 0.83 fold) in SP vs. Ctrl using DAVID. (A) Biological process, (B) Cellular Component, and (C) Molecular Function.

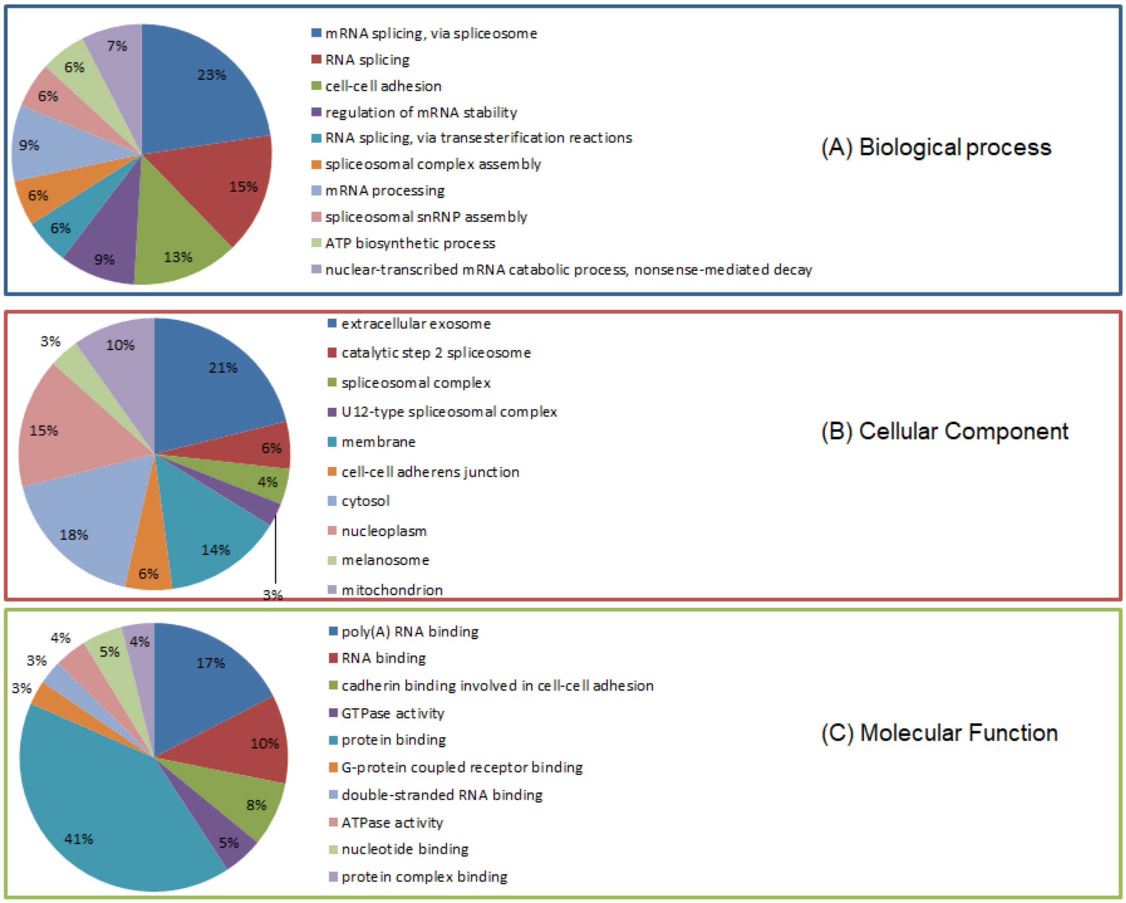

Figure S6. GO enrichment analysis of differentially expressed proteins (>1.25 fold or < 0.83 fold) in SP+Perindopril vs. SP using DAVID. (A) Biological process, (B) Cellular Component, and (C) Molecular Function.

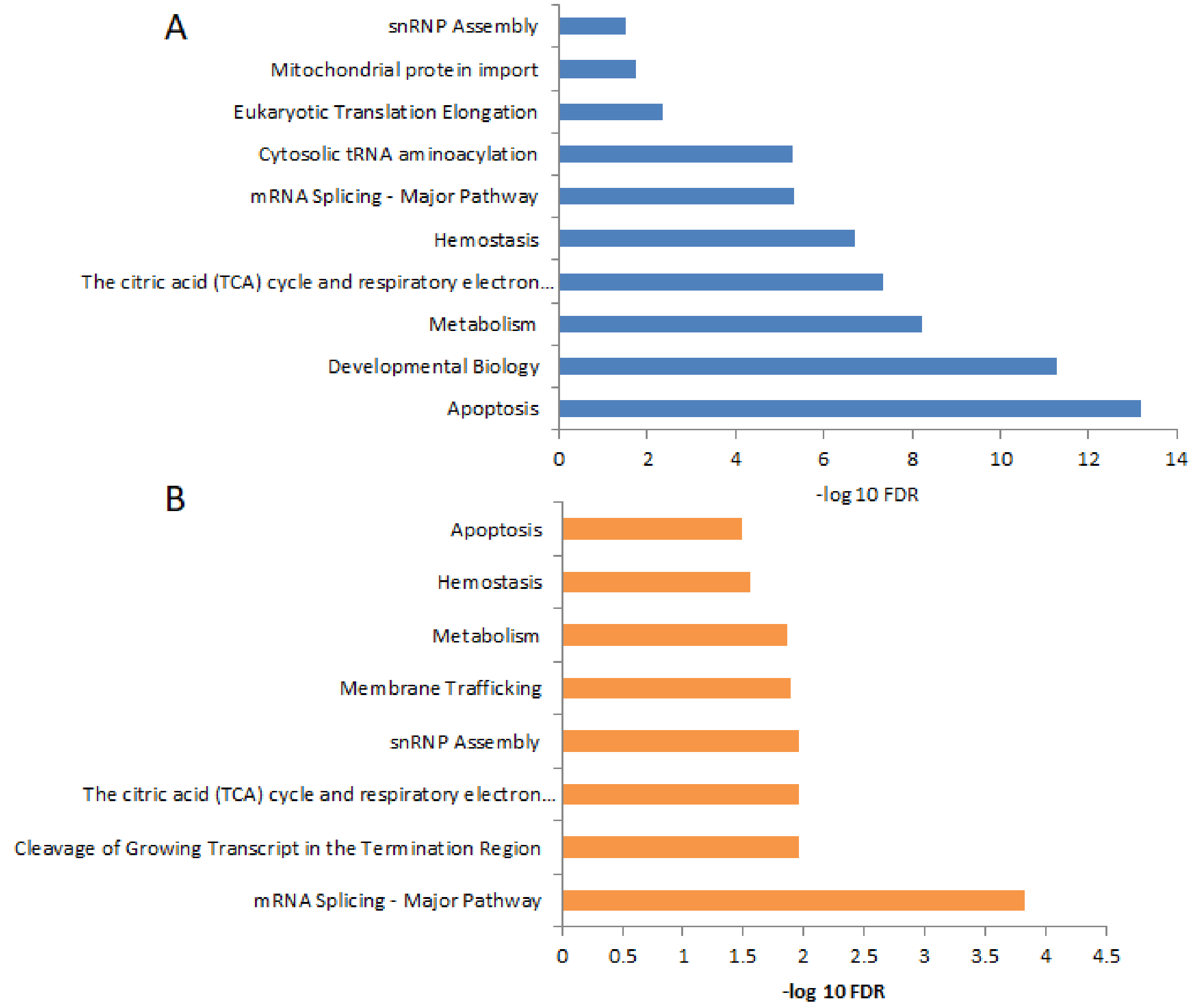

Figure S7. Reactome pathway analysis of differentially expressed proteins using STRING. Top Enrichment pathway classes for (A) differentially expressed proteins in SP vs. Ctrl, and (B) differentially expressed proteins in SP+Perindropil vs. SP. The enrichment analysis was set with statistical criteria ($p < 0.05$).

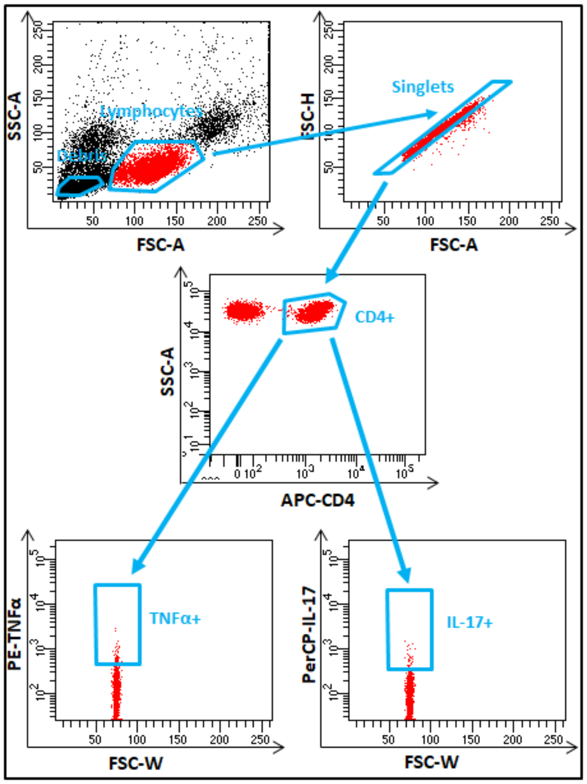

Figure S8. Gating strategies PBMCs. After staining with corresponding Abs, cells were analysed by flow cytometry. Fluorophores were respectively excited and analyzed with the appropriate laser and band pass filter (BP). The first gate was applied on the lymphocytes in the side scatter area (SSC-A)/forward scatter area (FSC-A) plot. Singlet population were gated using FSC-H over FSC-A. CD4+ T cells were gated in the SSC-A/CD4. TNFα+ and IL-17 T cells were gated in the plot from the CD4+ cells population.

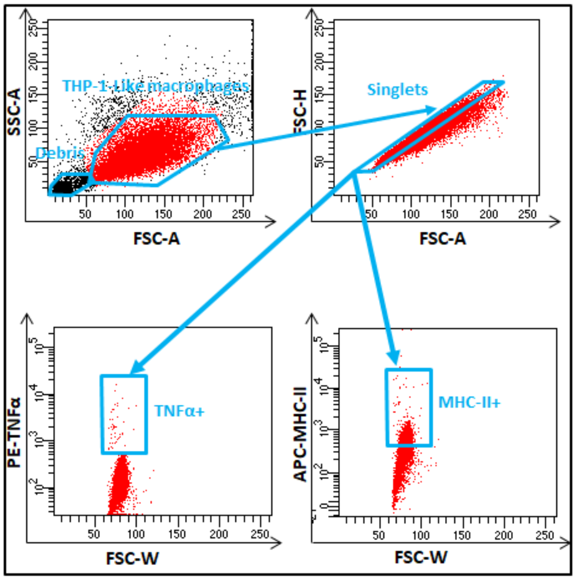

Figure S9. Gating strategies THP-1-Like macrophages. After staining with corresponding Abs, cells were analysed by flow cytometry. Fluorophores were respectively excited and analyzed with the appropriate laser and band pass filter (BP). The first gate was applied on the THP-1 like macrophages population after exclusion of debris in the side scatter area (SSC-A)/forward scatter area (FSC-A) plot. Singlet population were gated using FSC-H over FSC-A. TNFα+ and MHC-II positive cells were gated in the plot from the singlet cells population.

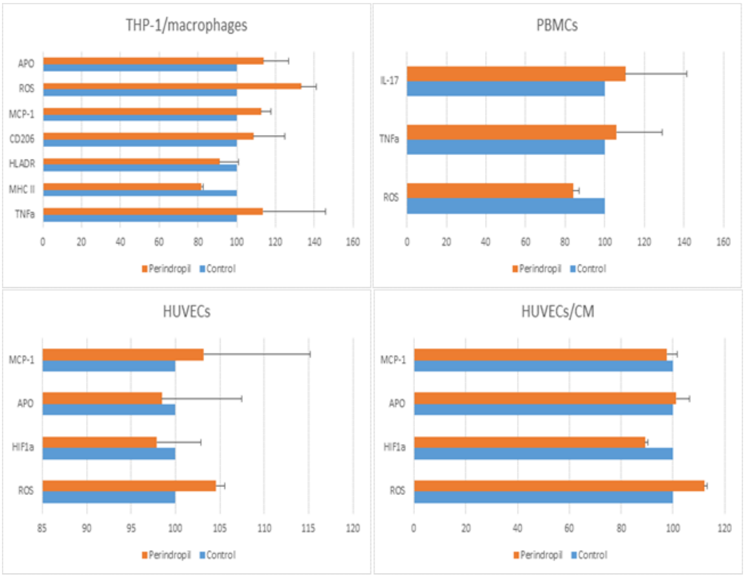

Figure S10. Cells were mock treated (Control) or pretreated with ACE inhibitor (Perindopril, 100 µM). Apoptosis, ROS, MCP-1, CD206, HLADR, MHCII, IL-17, TNFα and HIF1α were

evaluated in THP-1 macrophages, PBMCs and HUVECs using flow cytometry analysis. Data are presented as mean ± SEM, n=3-4.

Table S1.

Antibodies	Fluorochrome	Clone	Company
TNFα	PE	MAb11	Biolegend
MHC-II	APC	AF6-120.1	eBioscience
HLA-DR	Alexa Fluor 700	LN3	eBioscience
CD14	FITC	TuK4	eBioscience
CD206	PE	685641	eBioscience
CD4	APC	RM4-5	eBioscience
TNFα	PE	MAb11	Biolegend
CD4	PerCP	MEM-241	eBioscience
IL-17	FITC	eBio17B7	eBioscience
IL-17	PerCP	eBio64DEC17	eBioscience
INFg	FITC	4S.B3	Biolegend
CD56	PE-Cyanine7	CMSSB	eBioscience
INFg	FITC	4S.B3	Biolegend
CD8	APC-eFluor 780	RPA-T8	eBioscience
INFg	FITC	4S.B3	Biolegend
HIF1α	PE	546-16	Biolegend

PE, phycoerythrin; APC, allophycocyanin; FITC, Fluorescein.

Table S2.

PROTEIN	P value	Mean of Ctrl	Mean of SP	FC(SP/Ctrl)	Regulation

sp|P55327|TPD52_HUMAN	0.020184	33450	22575	0.67	Down
sp|O14745|NHRF1_HUMAN	0.019899	41350	45900	1.11	Up
sp|Q969H8|MYDGF_HUMAN	0.014596	28625	32275	1.13	Up
sp|P19105|ML12A_HUMAN	0.025541	154500	175750	1.14	Up
sp|O43399|TPD54_HUMAN	0.005861	26275	29975	1.14	Up
sp|P25398|RS12_HUMAN	0.003909	95275	108750	1.14	Up
sp|P61020|RAB5B_HUMAN	0.049623	47900	55200	1.15	Up
sp|P32119|PRDX2_HUMAN	0.002108	45350	52350	1.15	Up
sp|P61604|CH10_HUMAN	0.017942	648250	749500	1.16	Up
sp|P49321|NASP_HUMAN	0.010446	38500	44600	1.16	Up
sp|P55036|PSMD4_HUMAN	0.021255	26000	30200	1.16	Up
sp|P37802|TAGL2_HUMAN	0.030233	104350	121500	1.16	Up
sp|P52566|GDIR2_HUMAN	0.032515	403250	470250	1.17	Up
sp|Q562R1|ACTBL_HUMAN	0.001552	1955000	2280000	1.17	Up
sp|Q8NFI4|F10A5_HUMAN	0.022844	39775	46425	1.17	Up
sp|O15511|ARPC5_HUMAN	0.028346	69925	81800	1.17	Up
sp|Q92522|H1X_HUMAN	0.037917	109750	128500	1.17	Up
sp|P60174|TPIS_HUMAN	0.041357	89275	104625	1.17	Up
sp|P28066|PSA5_HUMAN	0.027103	55100	64650	1.17	Up
sp|P09211|GSTP1_HUMAN	0.026809	193750	228000	1.18	Up
sp|Q15102|PA1B3_HUMAN	0.002871	33825	39825	1.18	Up
sp|P52907|CAZA1_HUMAN	0.03207	55525	65375	1.18	Up
sp|P26885|FKBP2_HUMAN	0.021151	46900	55325	1.18	Up
sp|Q9BVC6|TM109_HUMAN	0.004517	68200	80475	1.18	Up
sp|Q92804|RBP56_HUMAN	0.024339	51050	60350	1.18	Up
sp|P61204|ARF3_HUMAN	0.013868	100450	118750	1.18	Up
sp|P30044|PRDX5_HUMAN	0.047466	81975	97175	1.19	Up
sp|P62310|LSM3_HUMAN	0.02092	41025	48650	1.19	Up
sp|Q9UBT2|SAE2_HUMAN	0.026943	20425	24250	1.19	Up
sp|P41252|SYIC_HUMAN	0.027028	38675	45925	1.19	Up
sp|P37837|TALDO_HUMAN	0.033508	60125	71650	1.19	Up
sp|P23528|COF1_HUMAN	0.043303	260500	310500	1.19	Up
sp|P31948|STIP1_HUMAN	0.006464	75900	90475	1.19	Up
sp|P62937|PPIA_HUMAN	0.00633	538500	643000	1.19	Up
sp|P35580|MYH10_HUMAN	0.028695	42475	50725	1.19	Up
sp|P26583|HMGB2_HUMAN	0.020178	35700	42650	1.19	Up
sp|P18124|RL7_HUMAN	0.008773	61150	73125	1.20	Up
sp|P62280|RS11_HUMAN	0.034445	46725	55900	1.20	Up
sp|P99999|CYC_HUMAN	0.00946	50025	59875	1.20	Up
sp|Q16543|CDC37_HUMAN	0.049747	59100	70750	1.20	Up
sp|O75223|GGCT_HUMAN	0.006951	47775	57200	1.20	Up

sp|P62241|RS8_HUMAN	0.036344	59600	71375	1.20	Up
sp|P27797|CALR_HUMAN	0.017765	133750	160500	1.20	Up
sp|P26368|U2AF2_HUMAN	0.011795	69500	83425	1.20	Up
sp|P09651|ROA1_HUMAN	0.035686	97250	116750	1.20	Up
sp|P62854|RS26_HUMAN	0.025021	34275	41175	1.20	Up
sp|P62750|RL23A_HUMAN	0.027329	106250	127750	1.20	Up
sp|P62258|1433E_HUMAN	0.038748	94500	113750	1.20	Up
sp|Q9Y536|PAL4A_HUMAN	0.013425	575250	692500	1.20	Up
sp|P14314|GLU2B_HUMAN	0.011105	77125	93000	1.21	Up
sp|P62913|RL11_HUMAN	0.037726	42150	50875	1.21	Up
sp|O60234|GMFG_HUMAN	0.030002	83550	100900	1.21	Up
sp|P00813|ADA_HUMAN	0.003678	92100	111250	1.21	Up
sp|Q15046|SYK_HUMAN	0.015992	60475	73075	1.21	Up
sp|P61353|RL27_HUMAN	0.004243	87225	105500	1.21	Up
sp|P25787|PSA2_HUMAN	0.020032	46950	56825	1.21	Up
sp|Q92945|FUBP2_HUMAN	0.006003	57550	69675	1.21	Up
sp|P11142|HSP7C_HUMAN	0.00369	191750	233000	1.22	Up
sp|P14174|MIF_HUMAN	0.003795	864500	1050500	1.22	Up
sp|P60660|MYL6_HUMAN	0.010441	98950	120250	1.22	Up
sp|P31946|1433B_HUMAN	0.020681	60500	73525	1.22	Up
sp|P14618|KPYM_HUMAN	0.002509	236000	287000	1.22	Up
sp|P15586|GNS_HUMAN	0.007947	63675	77450	1.22	Up
sp|Q14289|FAK2_HUMAN	0.033403	18675	22725	1.22	Up
sp|P01893|HLAH_HUMAN	0.03425	54300	66150	1.22	Up
sp|P63104|1433Z_HUMAN	0.044443	183750	224250	1.22	Up
sp|P16401|H15_HUMAN	0.040058	141750	173000	1.22	Up
sp|Q15365|PCBP1_HUMAN	0.007404	50100	61150	1.22	Up
sp|P62269|RS18_HUMAN	0.003155	92825	113500	1.22	Up
sp|P62910|RL32_HUMAN	0.044637	44700	54725	1.22	Up
sp|Q14103|HNRPD_HUMAN	0.007299	128000	156750	1.22	Up
sp|P18085|ARF4_HUMAN	0.039976	28675	35125	1.22	Up
sp|P47755|CAZA2_HUMAN	0.004802	251000	307750	1.23	Up
sp|P21399|ACOC_HUMAN	0.016599	37700	46250	1.23	Up
sp|Q99439|CNN2_HUMAN	0.008735	54625	67025	1.23	Up
sp|Q13435|SF3B2_HUMAN	0.048138	29500	36275	1.23	Up
sp|P25789|PSA4_HUMAN	0.010217	60400	74275	1.23	Up
sp|P63261|ACTG_HUMAN	0.019363	1185000	1457500	1.23	Up
sp|Q16555|DPYL2_HUMAN	0.007176	25975	31975	1.23	Up
sp|P35749|MYH11_HUMAN	0.014015	60475	74475	1.23	Up
sp|Q9UMX0|UBQL1_HUMAN	0.03988	27825	34275	1.23	Up
sp|Q7Z406|MYH14_HUMAN	0.046961	51825	63850	1.23	Up

sp|P62979|RS27A_HUMAN	0.008554	39525	48700	1.23	Up
sp|P42766|RL35_HUMAN	0.023666	38600	47575	1.23	Up
sp|P62736|ACTA_HUMAN	0.009313	1640000	2022500	1.23	Up
sp|P30040|ERP29_HUMAN	0.000699	79500	98225	1.24	Up
sp|Q13177|PAK2_HUMAN	0.012407	22575	27900	1.24	Up
sp|O75083|WDR1_HUMAN	0.005725	66125	81725	1.24	Up
sp|P68363|TBA1B_HUMAN	0.023302	352500	435750	1.24	Up
sp|P47756|CAPZB_HUMAN	0.007749	76875	95150	1.24	Up
sp|P60866|RS20_HUMAN	0.036143	38250	47350	1.24	Up
sp|P38646|GRP75_HUMAN	0.000432	47925	59375	1.24	Up
sp|Q13094|LCP2_HUMAN	0.000655	43350	53725	1.24	Up
sp|P12004|PCNA_HUMAN	0.014974	38100	47250	1.24	Up
sp|P49755|TMEDA_HUMAN	0.013991	42000	52100	1.24	Up
sp|P16402|H13_HUMAN	0.004055	109725	136250	1.24	Up
sp|Q96KP4|CNDP2_HUMAN	0.004715	34375	42725	1.24	Up
sp|P13693|TCTP_HUMAN	0.02907	77225	96000	1.24	Up
sp|P0CG47|UBB_HUMAN	0.033423	71625	89050	1.24	Up
sp|Q16777|H2A2C_HUMAN	0.018497	325250	404500	1.24	Up
sp|P30041|PRDX6_HUMAN	0.039182	51000	63450	1.24	Up
sp|P30626|SORCN_HUMAN	0.040951	40400	50275	1.24	Up
sp|P08133|ANXA6_HUMAN	0.002929	87775	109250	1.24	Up
sp|Q14141|SEPT6_HUMAN	0.003217	42825	53325	1.25	Up
sp|P00390|GSHR_HUMAN	0.045823	46725	58200	1.25	Up
sp|Q5JQF8|PAP1M_HUMAN	0.04128	47950	59750	1.25	Up
sp|O43707|ACTN4_HUMAN	0.009189	28725	35825	1.25	Up
sp|Q14240|IF4A2_HUMAN	0.002425	81250	101350	1.25	Up
sp|Q06830|PRDX1_HUMAN	0.001754	826250	1031000	1.25	Up
sp|P00558|PGK1_HUMAN	0.001543	230000	287000	1.25	Up
sp|P05141|ADT2_HUMAN	0.008095	80900	101050	1.25	Up
sp|P06733|ENOA_HUMAN	0.013236	220750	275750	1.25	Up
sp|Q13813|SPTN1_HUMAN	0.020835	48150	60150	1.25	Up
sp|O60831|PRAF2_HUMAN	0.007829	42200	52750	1.25	Up
sp|Q9BUJ2|HNRL1_HUMAN	0.021634	22450	28075	1.25	Up
sp|P61106|RAB14_HUMAN	0.000799	66300	82925	1.25	Up
sp|O60610|DIAP1_HUMAN	0.018176	42250	52850	1.25	Up
sp|P14550|AK1A1_HUMAN	0.008177	48825	61075	1.25	Up
sp|Q99638|RAD9A_HUMAN	0.026288	33150	41475	1.25	Up
sp|Q04917|1433F_HUMAN	0.013553	31075	38925	1.25	Up
sp|Q5TZA2|CROCC_HUMAN	0.001182	36800	46100	1.25	Up
sp|P07195|LDHB_HUMAN	0.003752	188750	236500	1.25	Up
sp|Q15631|TSN_HUMAN	0.002214	30400	38100	1.25	Up

sp|P57088|TMM33_HUMAN	0.022818	29450	36925	1.25	Up
sp|P68104|EF1A1_HUMAN	0.001142	285500	358000	1.25	Up
sp|Q15181|IPYR_HUMAN	0.000462	70250	88150	1.25	Up
sp|P35579|MYH9_HUMAN	0.002023	66050	82900	1.26	Up
sp|P24666|PPAC_HUMAN	0.003366	66000	82850	1.26	Up
sp|O14818|PSA7_HUMAN	0.044394	48550	60975	1.26	Up
sp|Q8IYT4|KATL2_HUMAN	0.000463	147250	185000	1.26	Up
sp|Q15907|RB11B_HUMAN	0.00025	25625	32200	1.26	Up
sp|P34932|HSP74_HUMAN	0.002554	46250	58125	1.26	Up
sp|P50991|TCPD_HUMAN	0.00942	52050	65425	1.26	Up
sp|P61077|UB2D3_HUMAN	0.00095	37925	47700	1.26	Up
sp|P63000|RAC1_HUMAN	0.000068	144500	181750	1.26	Up
sp|Q8WYJ6|SEPT1_HUMAN	0.047524	29725	37400	1.26	Up
sp|P13473|LAMP2_HUMAN	0.006916	51775	65150	1.26	Up
sp|Q12931|TRAP1_HUMAN	0.010954	428250	539000	1.26	Up
sp|P55060|XPO2_HUMAN	0.000791	29575	37225	1.26	Up
sp|P51665|PSMD7_HUMAN	0.00981	33150	41725	1.26	Up
sp|Q13509|TBB3_HUMAN	0.00254	473500	596250	1.26	Up
sp|P04406|G3P_HUMAN	0.042563	595250	749750	1.26	Up
sp|P26038|MOES_HUMAN	0.003357	149250	188000	1.26	Up
sp|Q9Y2B0|CNPY2_HUMAN	0.034199	51850	65325	1.26	Up
sp|O15533|TPSN_HUMAN	0.040079	36150	45550	1.26	Up
sp|P46776|RL27A_HUMAN	0.043189	66975	84450	1.26	Up
sp|Q7KZF4|SND1_HUMAN	0.014355	28250	35650	1.26	Up
sp|P22392|NDKB_HUMAN	0.005942	114900	145000	1.26	Up
sp|O14979|HNRDL_HUMAN	0.008302	172250	217500	1.26	Up
sp|P05455|LA_HUMAN	0.015547	21500	27150	1.26	Up
sp|P12956|XRCC6_HUMAN	0.001345	71700	90550	1.26	Up
sp|P54136|SYRC_HUMAN	0.000212	59525	75175	1.26	Up
sp|P31939|PUR9_HUMAN	0.01596	41200	52050	1.26	Up
sp|Q01844|EWS_HUMAN	0.030613	25525	32250	1.26	Up
sp|P48643|TCPE_HUMAN	0.003935	52650	66525	1.26	Up
sp|P08590|MYL3_HUMAN	0.032129	40925	51750	1.26	Up
sp|Q9UHX1|PUF60_HUMAN	0.002893	16700	21125	1.26	Up
sp|P59998|ARPC4_HUMAN	0.033217	59300	75025	1.27	Up
sp|Q86UX7|URP2_HUMAN	0.003292	36650	46375	1.27	Up
sp|P78417|GSTO1_HUMAN	0.001175	68075	86150	1.27	Up
sp|P68371|TBB4B_HUMAN	0.002926	108825	137750	1.27	Up
sp|P62306|RUXF_HUMAN	0.003699	38925	49275	1.27	Up
sp|Q5JXB2|UE2NL_HUMAN	0.018203	51500	65225	1.27	Up
sp|P52597|HNRPF_HUMAN	0.003044	66800	84650	1.27	Up

sp|P54652|HSP72_HUMAN	0.002051	314250	398250	1.27	Up
sp|Q96AE4|FUBP1_HUMAN	0.00903	58850	74600	1.27	Up
sp|P55072|TERA_HUMAN	0.000983	72800	92300	1.27	Up
sp|P15311|EZRI_HUMAN	0.000763	46250	58650	1.27	Up
sp|P04075|ALDOA_HUMAN	0.002053	131750	167250	1.27	Up
sp|P61247|RS3A_HUMAN	0.005861	68000	86400	1.27	Up
sp|P62888|RL30_HUMAN	0.005457	51125	64975	1.27	Up
sp|P50213|IDH3A_HUMAN	0.045355	40225	51150	1.27	Up
sp|P14324|FPPS_HUMAN	0.012718	50350	64075	1.27	Up
sp|P46940|IQGA1_HUMAN	0.003264	33350	42450	1.27	Up
sp|Q12905|ILF2_HUMAN	0.001692	37925	48275	1.27	Up
sp|P42677|RS27_HUMAN	0.022907	39275	50000	1.27	Up
sp|P62826|RAN_HUMAN	0.004213	208500	265750	1.27	Up
sp|Q07020|RL18_HUMAN	0.003375	187250	238750	1.28	Up
sp|Q92598|HS105_HUMAN	0.004021	41875	53400	1.28	Up
sp|P17174|AATC_HUMAN	0.001356	23575	30075	1.28	Up
sp|P07437|TBB5_HUMAN	0.003887	373500	476500	1.28	Up
sp|P08238|HS90B_HUMAN	0.006775	354250	452000	1.28	Up
sp|P11021|BIP_HUMAN	0.002685	97375	124250	1.28	Up
sp|P35637|FUS_HUMAN	0.032053	51175	65300	1.28	Up
sp|Q13162|PRDX4_HUMAN	0.000563	33775	43125	1.28	Up
sp|P78371|TCPB_HUMAN	0.031899	68175	87050	1.28	Up
sp|P13796|PLSL_HUMAN	0.003466	191000	244000	1.28	Up
sp|Q9NQH7|XPP3_HUMAN	0.004018	204500	261250	1.28	Up
sp|P21333|FLNA_HUMAN	0.000067	26125	33375	1.28	Up
sp|P18621|RL17_HUMAN	0.022341	1135000	1450000	1.28	Up
sp|P13010|XRCC5_HUMAN	0.002627	90200	115250	1.28	Up
sp|P61224|RAP1B_HUMAN	0.023074	48750	62325	1.28	Up
sp|Q71U36|TBA1A_HUMAN	0.016567	87200	111500	1.28	Up
sp|P31146|COR1A_HUMAN	0.002363	232250	297000	1.28	Up
sp|O00299|CLIC1_HUMAN	0.026669	99300	127000	1.28	Up
sp|Q9HB71|CYBP_HUMAN	0.002743	66075	84600	1.28	Up
sp|P27695|APEX1_HUMAN	0.00012	65950	84450	1.28	Up
sp|P30050|RL12_HUMAN	0.000442	53500	68525	1.28	Up
sp|P40121|CAPG_HUMAN	0.00675	47650	61075	1.28	Up
sp|O43776|SYNC_HUMAN	0.000618	31550	40450	1.28	Up
sp|P28072|PSB6_HUMAN	0.004021	52275	67025	1.28	Up
sp|P49773|HINT1_HUMAN	0.009119	23250	29825	1.28	Up
sp|P13639|EF2_HUMAN	0.004314	82675	106075	1.28	Up
sp|P62701|RS4X_HUMAN	0.018441	49000	62875	1.28	Up
sp|Q00839|HNRPU_HUMAN	0.026984	68125	87450	1.28	Up

sp|Q06323|PSME1_HUMAN	0.006073	66050	84825	1.28	Up
sp|Q15233|NONO_HUMAN	0.031465	51450	66075	1.28	Up
sp|Q12874|SF3A3_HUMAN	0.012181	28900	37125	1.28	Up
sp|P11413|G6PD_HUMAN	0.004484	60300	77475	1.28	Up
sp|P46926|GNPI1_HUMAN	0.006819	45100	57950	1.28	Up
sp|P15259|PGAM2_HUMAN	0.000667	141250	181500	1.28	Up
sp|P62834|RAP1A_HUMAN	0.017743	48175	61925	1.29	Up
sp|P60953|CDC42_HUMAN	0.017019	87275	112250	1.29	Up
sp|P36578|RL4_HUMAN	0.015913	54650	70325	1.29	Up
sp|P22314|UBA1_HUMAN	0.001872	60475	77875	1.29	Up
sp|P07741|APT_HUMAN	0.010178	58725	75675	1.29	Up
sp|P07900|HS90A_HUMAN	0.006409	140000	180500	1.29	Up
sp|Q9BZV1|UBXN6_HUMAN	0.033751	32100	41450	1.29	Up
sp|O75822|EIF3J_HUMAN	0.016186	86200	111325	1.29	Up
sp|P09972|ALDOC_HUMAN	0.002452	70500	91050	1.29	Up
sp|P22234|PUR6_HUMAN	0.005389	62750	81050	1.29	Up
sp|Q5BJF6|ODFP2_HUMAN	0.041616	34275	44275	1.29	Up
sp|P34897|GLYM_HUMAN	0.001409	54125	69925	1.29	Up
sp|O75874|IDHC_HUMAN	0.03841	39175	50625	1.29	Up
sp|P62316|SMD2_HUMAN	0.001086	30475	39425	1.29	Up
sp|O00231|PSD11_HUMAN	0.000119	42275	54700	1.29	Up
sp|P50990|TCPQ_HUMAN	0.000122	95425	123500	1.29	Up
sp|P52209|6PGD_HUMAN	0.001153	64475	83525	1.30	Up
sp|P61978|HNRPK_HUMAN	0.000727	84700	109750	1.30	Up
sp|P09622|DLDH_HUMAN	0.009371	37875	49100	1.30	Up
sp|P08865|RSSA_HUMAN	0.002301	76275	98975	1.30	Up
sp|P00492|HPRT_HUMAN	0.023888	37275	48375	1.30	Up
sp|Q9H2U2|IPYR2_HUMAN	0.007713	110350	143250	1.30	Up
sp|P55884|EIF3B_HUMAN	0.034795	54225	70400	1.30	Up
sp|P22307|NLTP_HUMAN	0.008648	38125	49500	1.30	Up
sp|P56537|IF6_HUMAN	0.001752	69775	90600	1.30	Up
sp|P08559|ODPA_HUMAN	0.016938	24925	32375	1.30	Up
sp|Q92930|RAB8B_HUMAN	0.013683	132750	172500	1.30	Up
sp|Q15286|RAB35_HUMAN	0.013683	132750	172500	1.30	Up
sp|P62820|RAB1A_HUMAN	0.000041	78875	102500	1.30	Up
sp|Q9UKA9|PTBP2_HUMAN	0.004613	49075	63775	1.30	Up
sp|P31153|METK2_HUMAN	0.011578	28025	36425	1.30	Up
sp|P49368|TCPG_HUMAN	0.002945	80500	104675	1.30	Up
sp|P62851|RS25_HUMAN	0.007241	77900	101325	1.30	Up
sp|P49207|RL34_HUMAN	0.000377	32150	41825	1.30	Up
sp|P61158|ARP3_HUMAN	0.000061	48175	62675	1.30	Up

sp|P25786|PSA1_HUMAN	0.008604	40450	52625	1.30	Up
sp|P60900|PSA6_HUMAN	0.00144	57375	74675	1.30	Up
sp|P08670|VIME_HUMAN	0.001791	144250	187750	1.30	Up
sp|P40227|TCPZ_HUMAN	0.002392	88875	115750	1.30	Up
sp|Q15029|U5S1_HUMAN	0.01184	108425	141250	1.30	Up
sp|Q14194|DPYL1_HUMAN	0.031375	29775	38800	1.30	Up
sp|P59190|RAB15_HUMAN	0.001975	144000	187750	1.30	Up
sp|P20340|RAB6A_HUMAN	0.001975	144000	187750	1.30	Up
sp|Q9H082|RB33B_HUMAN	0.001975	144000	187750	1.30	Up
sp|Q86V81|THOC4_HUMAN	0.008853	20200	26350	1.30	Up
sp|O75390|CISY_HUMAN	0.000135	61950	80825	1.30	Up
sp|P56134|ATPK_HUMAN	0.005344	68325	89175	1.31	Up
sp|P62318|SMD3_HUMAN	0.000797	105125	137250	1.31	Up
sp|P22626|ROA2_HUMAN	0.006737	66375	86700	1.31	Up
sp|P12081|SYHC_HUMAN	0.009644	135000	176500	1.31	Up
sp|P53396|ACLY_HUMAN	0.001367	44850	58650	1.31	Up
sp|Q9NY33|DPP3_HUMAN	0.00658	20800	27200	1.31	Up
sp|P61313|RL15_HUMAN	0.003806	30450	39825	1.31	Up
sp|P36871|PGM1_HUMAN	0.045403	26700	34925	1.31	Up
sp|Q02543|RL18A_HUMAN	0.011087	37025	48450	1.31	Up
sp|O43175|SERA_HUMAN	0.000179	29975	39225	1.31	Up
sp|P15153|RAC2_HUMAN	0.005935	72725	95175	1.31	Up
sp|P19623|SPEE_HUMAN	0.042662	35200	46075	1.31	Up
sp|Q16181|SEPT7_HUMAN	0.008633	32525	42575	1.31	Up
sp|P30049|ATPD_HUMAN	0.042263	36200	47400	1.31	Up
sp|Q9Y3I0|RTCB_HUMAN	0.015408	34500	45175	1.31	Up
sp|Q9UKX3|MYH13_HUMAN	0.002253	35100	45975	1.31	Up
sp|P23368|MAOM_HUMAN	0.002966	39025	51125	1.31	Up
sp|P62753|RS6_HUMAN	0.015135	80950	106050	1.31	Up
sp|P40926|MDHM_HUMAN	0.000339	162250	212750	1.31	Up
sp|P50914|RL14_HUMAN	0.015858	60925	79900	1.31	Up
sp|P62249|RS16_HUMAN	0.000705	77350	101450	1.31	Up
sp|P00491|PNPH_HUMAN	0.00096	122750	161000	1.31	Up
sp|P62273|RS29_HUMAN	0.02416	31875	41825	1.31	Up
sp|P06744|G6PI_HUMAN	0.004229	85925	112750	1.31	Up
sp|P55795|HNRH2_HUMAN	0.001006	42100	55275	1.31	Up
sp|P10809|CH60_HUMAN	0.00102	277000	363750	1.31	Up
sp|Q15084|PDIA6_HUMAN	0.005028	77250	101450	1.31	Up
sp|P30048|PRDX3_HUMAN	0.000903	110000	144500	1.31	Up
sp|P07237|PDIA1_HUMAN	0.007731	71850	94450	1.31	Up
sp|P35241|RADI_HUMAN	0.000264	265000	348500	1.32	Up

sp|P54727|RD23B_HUMAN	0.026999	39650	52150	1.32	Up
sp|P62244|RS15A_HUMAN	0.000061	36275	47725	1.32	Up
sp|P19087|GNAT2_HUMAN	0.003232	77700	102250	1.32	Up
sp|Q03113|GNA12_HUMAN	0.003232	77700	102250	1.32	Up
sp|P62424|RL7A_HUMAN	0.032319	49325	64925	1.32	Up
sp|Q01518|CAP1_HUMAN	0.000197	53750	70750	1.32	Up
sp|P62906|RL10A_HUMAN	0.000145	28925	38075	1.32	Up
sp|P11310|ACADM_HUMAN	0.018116	38075	50125	1.32	Up
sp|P62263|RS14_HUMAN	0.009163	48475	63825	1.32	Up
sp|P48735|IDHP_HUMAN	0.000166	50625	66700	1.32	Up
sp|P11940|PABP1_HUMAN	0.002478	39075	51500	1.32	Up
sp|Q6P2Q9|PRP8_HUMAN	0.026966	11910	15700	1.32	Up
sp|P62917|RL8_HUMAN	0.001178	19850	26175	1.32	Up
sp|Q9Y4L1|HYOU1_HUMAN	0.043441	25475	33600	1.32	Up
sp|Q9BWD1|THIC_HUMAN	0.010884	34750	45850	1.32	Up
sp|P39023|RL3_HUMAN	0.02404	16825	22200	1.32	Up
sp|Q12906|ILF3_HUMAN	0.025087	25000	33000	1.32	Up
sp|P05023|AT1A1_HUMAN	0.03313	28275	37325	1.32	Up
sp|P62491|RB11A_HUMAN	0.005842	72575	95850	1.32	Up
sp|O15144|ARPC2_HUMAN	0.005363	47375	62600	1.32	Up
sp|P26599|PTBP1_HUMAN	0.002471	40825	54000	1.32	Up
sp|Q13200|PSMD2_HUMAN	0.002685	20575	27225	1.32	Up
sp|P62873|GBB1_HUMAN	0.028253	39825	52700	1.32	Up
sp|Q15738|NSDHL_HUMAN	0.008687	26350	34875	1.32	Up
sp|P25788|PSA3_HUMAN	0.004242	74750	98950	1.32	Up
sp|Q01813|PFKAP_HUMAN	0.003252	31500	41700	1.32	Up
sp|P46782|RS5_HUMAN	0.007893	40575	53725	1.32	Up
sp|P61981|1433G_HUMAN	0.004224	59825	79225	1.32	Up
sp|P49448|DHE4_HUMAN	0.004793	54075	71625	1.32	Up
sp|P00338|LDHA_HUMAN	0.004812	267750	354750	1.32	Up
sp|P83881|RL36A_HUMAN	0.010682	25675	34025	1.33	Up
sp|P49411|EFTU_HUMAN	0.000729	39300	52100	1.33	Up
sp|P30613|KPYR_HUMAN	0.001261	613250	813500	1.33	Up
sp|Q9UHD8|SEPT9_HUMAN	0.014223	41650	55300	1.33	Up
sp|P04083|ANXA1_HUMAN	0.000109	78875	104725	1.33	Up
sp|P17980|PRS6A_HUMAN	0.002152	34250	45475	1.33	Up
sp|P29401|TKT_HUMAN	0.00821	59000	78350	1.33	Up
sp|O00154|BACH_HUMAN	0.005185	49700	66000	1.33	Up
sp|Q16563|SYPL1_HUMAN	0.010186	71100	94425	1.33	Up
sp|P08575|PTPRC_HUMAN	0.000717	63325	84150	1.33	Up
sp|O95861|BPNT1_HUMAN	0.00636	19300	25650	1.33	Up

sp|O75915|PRAF3_HUMAN	0.03958	57025	75850	1.33	Up
sp|P46781|RS9_HUMAN	0.000371	69175	92075	1.33	Up
sp|P12236|ADT3_HUMAN	0.002567	144000	191750	1.33	Up
sp|Q9Y3U8|RL36_HUMAN	0.01249	43200	57525	1.33	Up
sp|O15260|SURF4_HUMAN	0.009637	88400	117750	1.33	Up
sp|P28062|PSB8_HUMAN	0.000591	43225	57600	1.33	Up
sp|P34896|GLYC_HUMAN	0.00788	42875	57150	1.33	Up
sp|Q14761|PTCA_HUMAN	0.008274	35650	47525	1.33	Up
sp|P06576|ATPB_HUMAN	0.000396	128250	171000	1.33	Up
sp|P08195|4F2_HUMAN	0.00001	40850	54475	1.33	Up
sp|P07339|CATD_HUMAN	0.00112	87500	116750	1.33	Up
sp|P61254|RL26_HUMAN	0.031748	95925	128000	1.33	Up
sp|Q8TAA3|PSMA8_HUMAN	0.003399	58650	78325	1.34	Up
sp|Q9H4E7|DEFI6_HUMAN	0.002731	38300	51150	1.34	Up
sp|P07686|HEXB_HUMAN	0.012154	34750	46425	1.34	Up
sp|P40925|MDHC_HUMAN	0.001898	56450	75425	1.34	Up
sp|P04439|HLAA_HUMAN	0.000366	61300	81925	1.34	Up
sp|Q14697|GANAB_HUMAN	0.000681	34450	46050	1.34	Up
sp|P51991|ROA3_HUMAN	0.001801	36075	48225	1.34	Up
sp|P49721|PSB2_HUMAN	0.000315	52050	69650	1.34	Up
sp|P12268|IMDH2_HUMAN	0.002151	48500	64925	1.34	Up
sp|P31943|HNRH1_HUMAN	0.004487	39225	52600	1.34	Up
sp|P20700|LMNB1_HUMAN	0.033864	32100	43050	1.34	Up
sp|P23526|SAHH_HUMAN	0.002616	43525	58400	1.34	Up
sp|P63096|GNAI1_HUMAN	0.011376	35825	48125	1.34	Up
sp|Q15942|ZYX_HUMAN	0.015118	21875	29425	1.35	Up
sp|Q9UNM6|PSD13_HUMAN	0.000172	24475	32925	1.35	Up
sp|Q99832|TCPH_HUMAN	0.001666	64600	86950	1.35	Up
sp|P36542|ATPG_HUMAN	0.005307	51950	69925	1.35	Up
sp|Q04837|SSBP_HUMAN	0.001279	24475	32975	1.35	Up
sp|P30153|2AAA_HUMAN	0.033852	41375	55750	1.35	Up
sp|Q9P258|RCC2_HUMAN	0.005907	40175	54150	1.35	Up
sp|P62312|LSM6_HUMAN	0.000988	17100	23050	1.35	Up
sp|P14866|HNRPL_HUMAN	0.00497	26975	36375	1.35	Up
sp|P61586|RHOA_HUMAN	0.018369	86375	116500	1.35	Up
sp|Q15366|PCBP2_HUMAN	0.003766	71250	96175	1.35	Up
sp|P53621|COPA_HUMAN	0.007846	22550	30450	1.35	Up
sp|O43324|MCA3_HUMAN	0.000474	22325	30150	1.35	Up
sp|Q7L1Q6|BZW1_HUMAN	0.031533	45575	61550	1.35	Up
sp|Q92888|ARHG1_HUMAN	0.035084	20675	27925	1.35	Up
sp|Q16836|HCDH_HUMAN	0.037191	65850	89025	1.35	Up

sp|P18669|PGAM1_HUMAN	0.005208	69600	94125	1.35	Up
sp|Q3ZCM7|TBB8_HUMAN	0.002374	291500	394250	1.35	Up
sp|Q58FF3|ENPLL_HUMAN	0.003139	68400	92800	1.36	Up
sp|Q5SSJ5|HP1B3_HUMAN	0.018811	64825	87950	1.36	Up
sp|Q15393|SF3B3_HUMAN	0.034535	29425	39925	1.36	Up
sp|P41250|GARS_HUMAN	0.033901	29775	40400	1.36	Up
sp|P50395|GDIB_HUMAN	0.000431	53425	72525	1.36	Up
sp|P23284|PPIB_HUMAN	0.004586	37425	50850	1.36	Up
sp|P61160|ARP2_HUMAN	0.004726	63975	87000	1.36	Up
sp|P14625|ENPL_HUMAN	0.00507	101025	137500	1.36	Up
sp|P12814|ACTN1_HUMAN	0.000905	61000	83025	1.36	Up
sp|Q9UL46|PSME2_HUMAN	0.021743	42175	57425	1.36	Up
sp|P31930|QCR1_HUMAN	0.001525	33925	46200	1.36	Up
sp|Q14568|HS902_HUMAN	0.008177	245250	334000	1.36	Up
sp|Q13838|DX39B_HUMAN	0.000016	58100	79150	1.36	Up
sp|Q6S8J3|POTEE_HUMAN	0.001765	581000	791500	1.36	Up
sp|P62277|RS13_HUMAN	0.006358	48500	66075	1.36	Up
sp|P07602|SAP_HUMAN	0.007114	92825	126500	1.36	Up
sp|P00367|DHE3_HUMAN	0.000617	32750	44675	1.36	Up
sp|Q14651|PLSI_HUMAN	0.004961	75150	102675	1.37	Up
sp|P46777|RL5_HUMAN	0.00128	40925	55950	1.37	Up
sp|Q9HAV0|GBB4_HUMAN	0.036688	66050	90300	1.37	Up
sp|Q92499|DDX1_HUMAN	0.01882	28475	38950	1.37	Up
sp|P13667|PDIA4_HUMAN	0.001343	74750	102275	1.37	Up
sp|P51149|RAB7A_HUMAN	0.000148	47100	64500	1.37	Up
sp|Q9Y265|RUVB1_HUMAN	0.015229	44050	60325	1.37	Up
sp|Q99714|HCD2_HUMAN	0.000657	36325	49750	1.37	Up
sp|Q92841|DDX17_HUMAN	0.003291	37425	51275	1.37	Up
sp|P23396|RS3_HUMAN	0.001058	79250	108675	1.37	Up
sp|Q02790|FKBP4_HUMAN	0.013057	57950	79500	1.37	Up
sp|P25705|ATPA_HUMAN	0.001071	111150	152500	1.37	Up
sp|P15880|RS2_HUMAN	0.002644	47425	65125	1.37	Up
sp|O75533|SF3B1_HUMAN	0.000161	37175	51050	1.37	Up
sp|Q15459|SF3A1_HUMAN	0.009428	37700	51775	1.37	Up
sp|P29218|IMPA1_HUMAN	0.012578	39050	53725	1.38	Up
sp|P17066|HSP76_HUMAN	0.0068	448000	616750	1.38	Up
sp|Q13011|ECH1_HUMAN	0.000855	62875	86750	1.38	Up
sp|Q8IZL9|CDK20_HUMAN	0.016403	31050	42950	1.38	Up
sp|Q13404|UB2V1_HUMAN	0.002229	66525	92075	1.38	Up
sp|Q9NTK5|OLA1_HUMAN	0.008736	22175	30700	1.38	Up
sp|Q08043|ACTN3_HUMAN	0.005276	62375	86375	1.38	Up

sp|A6NHL2|TBAL3_HUMAN	0.0107	367250	508750	1.39	Up
sp|P48556|PSMD8_HUMAN	0.019684	32925	45625	1.39	Up
sp|Q6NVV1|R13P3_HUMAN	0.001105	62875	87275	1.39	Up
sp|P61163|ACTZ_HUMAN	0.049632	65125	90400	1.39	Up
sp|Q9NVJ2|ARL8B_HUMAN	0.039069	39375	54675	1.39	Up
sp|Q8NHW5|RLA0L_HUMAN	0.00036	58625	81550	1.39	Up
sp|Q9H9B4|SFXN1_HUMAN	0.019787	29825	41525	1.39	Up
sp|P15121|ALDR_HUMAN	0.038192	116850	163000	1.39	Up
sp|P38117|ETFB_HUMAN	0.045982	25975	36250	1.40	Up
sp|P63244|RACK1_HUMAN	0.020312	110500	154250	1.40	Up
sp|P32969|RL9_HUMAN	0.007724	56375	78750	1.40	Up
sp|P26641|EF1G_HUMAN	0.002077	67125	94125	1.40	Up
sp|P62314|SMD1_HUMAN	0.005686	45850	64300	1.40	Up
sp|P54577|SYYC_HUMAN	0.000775	16450	23100	1.40	Up
sp|P14868|SYDC_HUMAN	0.037338	45300	63675	1.41	Up
sp|Q00610|CLH1_HUMAN	0.003894	36525	51350	1.41	Up
sp|P04899|GNAI2_HUMAN	0.002889	20425	28775	1.41	Up
sp|O15145|ARPC3_HUMAN	0.034526	101500	143000	1.41	Up
sp|O14744|ANM5_HUMAN	0.000104	42300	59625	1.41	Up
sp|O43684|BUB3_HUMAN	0.0014	19825	27975	1.41	Up
sp|P48047|ATPO_HUMAN	0.000269	49500	69850	1.41	Up
sp|P06865|HEXA_HUMAN	0.018022	32275	45550	1.41	Up
sp|O75964|ATP5L_HUMAN	0.000152	47075	66450	1.41	Up
sp|P09661|RU2A_HUMAN	0.000239	25600	36175	1.41	Up
sp|P00505|AATM_HUMAN	0.045609	78450	111025	1.42	Up
sp|P22695|QCR2_HUMAN	0.001547	48075	68075	1.42	Up
sp|Q9NYU2|UGGG1_HUMAN	0.032258	24650	34925	1.42	Up
sp|Q9NPJ3|ACO13_HUMAN	0.010647	40200	57000	1.42	Up
sp|P12931|SRC_HUMAN	0.003111	24250	34400	1.42	Up
sp|P02656|APOC3_HUMAN	0.000071	17800	25375	1.43	Up
sp|P00846|ATP6_HUMAN	0.012047	38475	54950	1.43	Up
sp|Q02878|RL6_HUMAN	0.008175	56100	80150	1.43	Up
sp|Q9NSD9|SYFB_HUMAN	0.002013	26950	38525	1.43	Up
sp|Q96HE7|ERO1A_HUMAN	0.015765	20325	29125	1.43	Up
sp|Q00534|CDK6_HUMAN	0.003112	40675	58325	1.43	Up
sp|P24539|AT5F1_HUMAN	0.001351	19225	27625	1.44	Up
sp|Q9Y4G6|TLN2_HUMAN	0.03231	59325	85300	1.44	Up
sp|Q08211|DHX9_HUMAN	0.008726	15850	22800	1.44	Up
sp|P55084|ECHB_HUMAN	0.008061	20075	28900	1.44	Up
sp|Q9UFN0|NPS3A_HUMAN	0.006674	20625	29700	1.44	Up
sp|P17844|DDX5_HUMAN	0.011018	22175	32075	1.45	Up

PROTEIN	P value	Mean of SP Perindropil	Mean of SP	FC(SP Perindropil /SP)	Regulation
sp|P30101|PDIA3_HUMAN	0.00144	75250	109000	1.45	Up
sp|P43403|ZAP70_HUMAN	0.000791	21825	31625	1.45	Up
sp|O00151|PDLI1_HUMAN	0.022387	64325	93625	1.46	Up
sp|P55212|CASP6_HUMAN	0.006715	17875	26050	1.46	Up
sp|P10768|ESTD_HUMAN	0.000173	16100	23500	1.46	Up
sp|P40939|ECHA_HUMAN	0.008758	23325	34050	1.46	Up
sp|Q13867|BLMH_HUMAN	0.003162	36000	52925	1.47	Up
sp|Q13263|TIF1B_HUMAN	0.003324	26300	38675	1.47	Up
sp|O15143|ARC1B_HUMAN	0.000038	25650	37800	1.47	Up
sp|Q9BRG1|VPS25_HUMAN	0.014642	27425	40425	1.47	Up
sp|P20645|MPRD_HUMAN	0.006667	27175	40125	1.48	Up
sp|P48449|ERG7_HUMAN	0.031039	35250	52050	1.48	Up
sp|P61326|MGN_HUMAN	0.00069	16500	24375	1.48	Up
sp|P0DMV9|HS71B_HUMAN	0.019727	25750	38150	1.48	Up
sp|P28070|PSB4_HUMAN	0.016788	102900	153750	1.49	Up
sp|Q9Y3A6|TMED5_HUMAN	0.031018	50100	75025	1.50	Up
sp|P07814|SYEP_HUMAN	0.021774	30025	45125	1.50	Up
sp|P27701|CD82_HUMAN	0.014487	52000	78375	1.51	Up
sp|Q00535|CDK5_HUMAN	0.007967	36450	55100	1.51	Up
sp|O00410|IPO5_HUMAN	0.014886	26550	40825	1.54	Up
sp|O95399|UTS2_HUMAN	0.016233	94875	146750	1.55	Up
sp|P51572|BAP31_HUMAN	0.012517	23625	36650	1.55	Up
sp|Q13303|KCAB2_HUMAN	0.021265	21175	33000	1.56	Up
sp|Q96C01|F136A_HUMAN	0.025	14138	22275	1.58	Up
sp|P42574|CASP3_HUMAN	0.023601	46475	74200	1.60	Up
sp|P54707|AT12A_HUMAN	0.01869	30100	48200	1.60	Up
sp|P26640|SYVC_HUMAN	0.013437	18675	30250	1.62	Up
sp|P23246|SFPQ_HUMAN	0.018717	38025	63925	1.68	Up
sp|P54578|UBP14_HUMAN	0.027376	38375	65025	1.69	Up
sp|P00387|NB5R3_HUMAN	0.019486	18125	31375	1.73	Up
sp|O43488|ARK72_HUMAN	0.003485	28750	49775	1.73	Up
sp|P24752|THIL_HUMAN	0.026228	52525	91025	1.73	Up
sp|Q2VIR3|IF2GL_HUMAN	0.037444	40750	72525	1.78	Up
sp|Q9BXJ9|NAA15_HUMAN	0.001933	18000	34750	1.93	Up
sp|Q13561|DCTN2_HUMAN	0.040456	20600	41575	2.02	Up
sp|Q01650|LAT1_HUMAN	0.027839	14350	29125	2.03	Up
sp|P09104|ENOG_HUMAN	0.019769	11458	24775	2.16	Up
sp|Q9HB07|MYG1_HUMAN	0.029104	48270	109550	2.27	Up
sp|Q92974|ARHG2_HUMAN	0.001286	3438	9773	2.84	Up

sp|P61088|UBE2N_HUMAN	0.026203	34475	66100	0.52	Down
sp|O43143|DHX15_HUMAN	0.005172	22825	40800	0.56	Down
sp|O75323|NIPS2_HUMAN	0.022578	6963	12085	0.58	Down
sp|P00387|NB5R3_HUMAN	0.031836	19200	31375	0.61	Down
sp|Q9BXJ9|NAA15_HUMAN	0.016941	22400	34750	0.64	Down
sp|Q9BYZ2|LDH6B_HUMAN	0.027255	42750	63975	0.67	Down
sp|Q96C01|F136A_HUMAN	0.012656	14900	22275	0.67	Down
sp|Q00535|CDK5_HUMAN	0.012953	38075	55100	0.69	Down
sp|P62314|SMD1_HUMAN	0.011325	44500	64300	0.69	Down
sp|Q9UHG3|PCYOX_HUMAN	0.008629	14500	19775	0.73	Down
sp|O43488|ARK72_HUMAN	0.009555	37000	49775	0.74	Down
sp|P62834|RAP1A_HUMAN	0.022908	46075	61925	0.74	Down
sp|O60664|PLIN3_HUMAN	0.02677	14975	19750	0.76	Down
sp|Q96HE7|ERO1A_HUMAN	0.028989	22125	29125	0.76	Down
sp|P20700|LMNB1_HUMAN	0.011465	32800	43050	0.76	Down
sp|Q6P2Q9|PRP8_HUMAN	0.009844	11975	15700	0.76	Down
sp|P56134|ATPK_HUMAN	0.009355	68325	89175	0.77	Down
sp|P07602|SAP_HUMAN	0.026199	97200	126500	0.77	Down
sp|Q8NHW5|RLA0L_HUMAN	0.049188	63000	81550	0.77	Down
sp|P49721|PSB2_HUMAN	0.026338	54300	69650	0.78	Down
sp|P31930|QCR1_HUMAN	0.015315	36200	46200	0.78	Down
sp|O00231|PSD11_HUMAN	0.016524	42975	54700	0.79	Down
sp|Q92930|RAB8B_HUMAN	0.033879	135750	172500	0.79	Down
sp|Q15286|RAB35_HUMAN	0.033879	135750	172500	0.79	Down
sp|P57088|TMM33_HUMAN	0.032687	29100	36925	0.79	Down
sp|Q15738|NSDHL_HUMAN	0.018936	27650	34875	0.79	Down
sp|Q9UKX3|MYH13_HUMAN	0.041036	36550	45975	0.79	Down
sp|Q13838|DX39B_HUMAN	0.019997	63025	79150	0.80	Down
sp|Q16563|SYPL1_HUMAN	0.01186	75350	94425	0.80	Down
sp|O43399|TPD54_HUMAN	0.042351	23925	29975	0.80	Down
sp|P22234|PUR6_HUMAN	0.024006	64800	81050	0.80	Down
sp|Q15393|SF3B3_HUMAN	0.012532	31925	39925	0.80	Down
sp|O75964|ATP5L_HUMAN	0.004536	53250	66450	0.80	Down
sp|P43403|ZAP70_HUMAN	0.019209	25375	31625	0.80	Down
sp|P51991|ROA3_HUMAN	0.010459	38700	48225	0.80	Down
sp|A8MWD9|RUXGL_HUMAN	0.044147	35000	43575	0.80	Down
sp|P30153|2AAA_HUMAN	0.038786	44850	55750	0.80	Down
sp|P02656|APOC3_HUMAN	0.004278	20425	25375	0.80	Down
sp|Q96KP4|CNDP2_HUMAN	0.016208	34475	42725	0.81	Down
sp|Q9UMX0|UBQL1_HUMAN	0.019156	27700	34275	0.81	Down
sp|Q92945|FUBP2_HUMAN	0.031433	56425	69675	0.81	Down

sp|P19087|GNAT2_HUMAN	0.04139	82825	102250	0.81	Down
sp|Q03113|GNA12_HUMAN	0.04139	82825	102250	0.81	Down
sp|P35637|FUS_HUMAN	0.036354	53100	65300	0.81	Down
sp|P54707|AT12A_HUMAN	0.025463	39350	48200	0.82	Down
sp|P61326|MGN_HUMAN	0.03915	19900	24375	0.82	Down
sp|P07741|APT_HUMAN	0.033908	61900	75675	0.82	Down
sp|Q9UKA9|PTBP2_HUMAN	0.043138	52200	63775	0.82	Down
sp|P20645|MPRD_HUMAN	0.011058	32875	40125	0.82	Down
sp|Q13263|TIF1B_HUMAN	0.045133	31750	38675	0.82	Down
sp|P31153|METK2_HUMAN	0.049977	29925	36425	0.82	Down
sp|P52597|HNRPF_HUMAN	0.043026	69550	84650	0.82	Down
sp|P04083|ANXA1_HUMAN	0.016374	86050	104725	0.82	Down
sp|P08195|4F2_HUMAN	0.034611	44775	54475	0.82	Down
sp|O75874|IDHC_HUMAN	0.000872	41675	50625	0.82	Down
sp|P62318|SMD3_HUMAN	0.036552	113000	137250	0.82	Down
sp|P0DMV9|HS71B_HUMAN	0.046796	31475	38150	0.83	Down
sp|O95433|AHSA1_HUMAN	0.042249	37875	45900	0.83	Down
sp|P61160|ARP2_HUMAN	0.041775	72075	87000	0.83	Down
sp|Q00610|CLH1_HUMAN	0.031672	42575	51350	0.83	Down
sp|P11940|PABP1_HUMAN	0.043652	42700	51500	0.83	Down
sp|P15586|GNS_HUMAN	0.048381	64275	77450	0.83	Down
sp|O14744|ANM5_HUMAN	0.027136	49525	59625	0.83	Down
sp|P49207|RL34_HUMAN	0.010547	34750	41825	0.83	Down
sp|P54727|RD23B_HUMAN	0.013349	43575	52150	0.84	Down
sp|P38117|ETFB_HUMAN	0.025777	30350	36250	0.84	Down
sp|Q9UNM6|PSD13_HUMAN	0.018704	27575	32925	0.84	Down
sp|P59190|RAB15_HUMAN	0.035502	157250	187750	0.84	Down
sp|P20340|RAB6A_HUMAN	0.035502	157250	187750	0.84	Down
sp|Q9H082|RB33B_HUMAN	0.035502	157250	187750	0.84	Down
sp|Q99638|RAD9A_HUMAN	0.006413	34800	41475	0.84	Down
sp|Q13404|UB2V1_HUMAN	0.047271	77300	92075	0.84	Down
sp|Q99832|TCPH_HUMAN	0.033126	73050	86950	0.84	Down
sp|P23396|RS3_HUMAN	0.047687	91325	108675	0.84	Down
sp|P61163|ACTZ_HUMAN	0.016537	76200	90400	0.84	Down
sp|P35241|RADI_HUMAN	0.047786	294500	348500	0.85	Down
sp|P62312|LSM6_HUMAN	0.033512	19500	23050	0.85	Down
sp|P22307|NLTP_HUMAN	0.048565	41900	49500	0.85	Down
sp|O00151|PDLI1_HUMAN	0.029886	79300	93625	0.85	Down
sp|P27695|APEX1_HUMAN	0.016459	71600	84450	0.85	Down
sp|P06744|G6PI_HUMAN	0.045021	95650	112750	0.85	Down
sp|P34897|GLYM_HUMAN	0.036804	59425	69925	0.85	Down

sp|P13473|LAMP2_HUMAN	0.045484	55375	65150	0.85	Down
sp|P42574|CASP3_HUMAN	0.035233	63150	74200	0.85	Down
sp|P15259|PGAM2_HUMAN	0.020282	154500	181500	0.85	Down
sp|Q13011|ECH1_HUMAN	0.013957	73875	86750	0.85	Down
sp|P61158|ARP3_HUMAN	0.045111	53500	62675	0.85	Down
sp|P10809|CH60_HUMAN	0.03977	310750	363750	0.85	Down
sp|P26599|PTBP1_HUMAN	0.044933	46150	54000	0.85	Down
sp|P62316|SMD2_HUMAN	0.026411	33700	39425	0.85	Down
sp|P22695|QCR2_HUMAN	0.000439	58650	68075	0.86	Down
sp|P40926|MDHM_HUMAN	0.027545	183500	212750	0.86	Down
sp|P23526|SAHH_HUMAN	0.014139	50500	58400	0.86	Down
sp|P56537|IF6_HUMAN	0.033144	78600	90600	0.87	Down
sp|P28062|PSB8_HUMAN	0.045977	49975	57600	0.87	Down
sp|P12931|SRC_HUMAN	0.041475	29850	34400	0.87	Down
sp|P61204|ARF3_HUMAN	0.015619	103275	118750	0.87	Down
sp|P21333|FLNA_HUMAN	0.033531	29075	33375	0.87	Down
sp|P61981|1433G_HUMAN	0.030683	69050	79225	0.87	Down
sp|Q9UI08|EVL_HUMAN	0.045528	30725	35050	0.88	Down
sp|P62906|RL10A_HUMAN	0.044058	33525	38075	0.88	Down
sp|Q07020|RL18_HUMAN	0.04986	211500	238750	0.89	Down
sp|P61604|CH10_HUMAN	0.025049	664250	749500	0.89	Down

PROTEIN	P value	Mean of Ctrl	Mean of SP Perindropil	FC(SP Perindropil /Ctrl)	Regulation
sp|Q99733|NP1L4_HUMAN	0.004228	47425	26475	0.56	Down
sp|O43143|DHX15_HUMAN	0.022877	32100	22825	0.71	Down
sp|Q16531|DDB1_HUMAN	0.033183	29000	22525	0.78	Down
sp|P18124|RL7_HUMAN	0.041507	61150	68700	1.12	Up
sp|Q13404|UB2V1_HUMAN	0.038442	66525	77300	1.16	Up
sp|Q13011|ECH1_HUMAN	0.041424	62875	73875	1.17	Up
sp|Q58FF3|ENPLL_HUMAN	0.041367	68400	80825	1.18	Up
sp|P07237|PDIA1_HUMAN	0.048115	71850	85150	1.19	Up
sp|P49448|DHE4_HUMAN	0.046826	54075	65275	1.21	Up
sp|P23368|MAOM_HUMAN	0.021259	39025	47575	1.22	Up
sp|P22695|QCR2_HUMAN	0.028807	48075	58650	1.22	Up
sp|O43765|SGTA_HUMAN	0.00291	20925	25700	1.23	Up
sp|P13667|PDIA4_HUMAN	0.025407	74750	92050	1.23	Up
sp|P00367|DHE3_HUMAN	0.011623	32750	40675	1.24	Up
sp|P10768|ESTD_HUMAN	0.029788	16100	20100	1.25	Up
sp|Q04837|SSBP_HUMAN	0.0045	24475	30675	1.25	Up
sp|P23284|PPIB_HUMAN	0.025136	37425	47050	1.26	Up
sp|Q14568|HS902_HUMAN	0.049335	245250	309250	1.26	Up

sp|Q96QK1|VPS35_HUMAN	0.045626	22300	28350	1.27	Up
sp|Q14194|DPYL1_HUMAN	0.045244	29775	38525	1.29	Up
sp|P06865|HEXA_HUMAN	0.038395	32275	42100	1.30	Up
sp|Q5SSJ5|HP1B3_HUMAN	0.040335	64825	85175	1.31	Up
sp|O15143|ARC1B_HUMAN	0.019623	25650	34050	1.33	Up
sp|Q13867|BLMH_HUMAN	0.043999	36000	48025	1.33	Up
sp|Q9H9B4|SFXN1_HUMAN	0.029645	29825	39950	1.34	Up
sp|P84103|SRSF3_HUMAN	0.031289	51775	72625	1.40	Up
sp|P07686|HEXB_HUMAN	0.001014	34750	51825	1.49	Up
sp|Q96RU3|FNBP1_HUMAN	0.00596	23975	36400	1.52	Up
sp|A6NEC2|PSAL_HUMAN	0.039362	22200	34200	1.54	Up
sp|O95399|UTS2_HUMAN	0.017679	94875	148250	1.56	Up
sp|Q9BWM7|SFXN3_HUMAN	0.006165	48975	84550	1.73	Up
sp|Q13561|DCTN2_HUMAN	0.001942	20600	40750	1.98	Up
sp|P35998|PRS7_HUMAN	0.021013	28900	69000	2.39	Up
sp|P38919|IF4A3_HUMAN	0.03199	29025	75475	2.60	Up

SP: S protein; SP Perindropil (SP with ACE inhibitor)
